# Comparative Characterization of Pork Jowl and Three Commercial Cuts from DLY Pigs: Instrumental Quality, Nutritional Composition, Water Distribution, and Volatile Profiles

**DOI:** 10.3390/foods15162823

**Published:** 2026-08-13

**Authors:** Shuanshuan Xue, Wanli Zhang, Jiaqi Sun, Bing Yang, Yingjian Hou, Minnan Liu, Zhenxia Cao, Jing Yan, Heng Wang, Lishui Chen

**Affiliations:** Food Laboratory of Zhong Yuan, Luohe 462300, China; xueshuanshuan@zyfoodlab.cn (S.X.); zhangwanli@zyfoodlab.cn (W.Z.); caozhenxia@zyfoodlab.cn (Z.C.); yanjing@zyfoodlab.cn (J.Y.)

**Keywords:** pork jowl, commercial pork cuts, DLY pigs, instrumental quality, amino-acid composition, volatile profile

## Abstract

This preliminary within-animal study focuses on the comprehensive characterization of pork jowl (PJ) by comparing it with pork belly (PB), Boston butt (BB), and spare ribs (SR) collected from the same ten castrated Duroc × Landrace × Yorkshire (DLY) pigs; headspace volatile analysis used a matched subset of six pigs. Instrumental quality, proximate composition, amino-acid and fatty-acid profiles, low-field nuclear magnetic resonance (LF-NMR) water distribution and headspace solid-phase microextraction–gas chromatography–mass spectrometry (HS-SPME-GC-MS) volatile profiles were evaluated. PB exhibited the lowest shear force, hardness, and chewiness. Across the four pork cuts, crude protein content ranged from 16.87% to 18.10%, whereas crude fat content ranged from 13.03% to 22.20%. BB had the lowest crude fat content (13.03%) and relatively high protein (18.10%) and amino-acid contents. PJ showed highest content of monounsaturated fatty acids (MUFAs) and the lowest numerical atherogenic (AI) and thrombogenic indices (TI), but also the highest n-6/n-3 polyunsaturated fatty acids (PUFA) ratio. LF-NMR showed that PJ had the highest T_23_ and P23 values, consistent with its higher cooking and centrifugal losses. Sixty-five headspace volatile compounds were tentatively identified under the standardized 80 °C HS-SPME conditions. OPLS-DA differentiated the four cuts, and ten compounds meeting the combined criteria of VIP > 1, q < 0.05, and OAV > 1 were retained as candidate discriminant aroma-relevant compounds. Overall, the four cuts exhibited distinct quality profiles: PJ combined a MUFA-rich lipid fraction with greater free-water mobility and water loss, PB showed the greatest instrumental tenderness, BB had the lowest crude fat content and relatively high protein and amino-acid contents, and SR exhibited the greatest headspace volatile abundance. The data provide a reference for cut-specific utilization of pork raw materials.

## 1. Introduction

Pork is a staple meat and an important source of animal protein in China. Duroc × Landrace × Yorkshire (DLY) crossbred pigs are widely used in commercial pork production because of their rapid growth and high lean-meat yield [1]. The rapid expansion of the prepared meat industry has shifted processing from empirical raw material selection toward standardized, data-driven approaches. Meat processors increasingly require quantitative, cut-specific quality indicators—including texture, nutritional composition, and volatile flavor profiles—to match raw materials to targeted product categories and optimize processing parameters [2,3,4,5]. However, quality data for under-characterized cuts such as pork jowl (PJ) remain insufficient, limiting evidence-based raw material selection and product development.

Pork quality is comprehensively evaluated from three interrelated dimensions: eating quality (water-holding capacity, tenderness, and color), nutritional composition (crude protein, fat, fatty-acid profiles, and amino-acid balance), and volatile flavor characteristics [3,6]. Previous studies have associated a higher proportion of glycolytic fibers with faster post mortem metabolism, lighter color, and greater drip loss, whereas relationships with tenderness and cooking loss are less consistent [7,8,9]. These findings indicate that muscle fiber type is not an independent predictor of pork quality but interacts with fiber size, connective tissue, fat deposition, genotype, and muscle location. Because PJ is a heterogeneous cervical muscle–adipose complex and its fiber composition was not measured here, muscle fiber characteristics are considered only as a possible biological context for the observed cut-related differences [7,8,9].

PJ is a relatively under-characterized pork cut containing naturally associated muscle and adipose tissues. In the present study, PJ was operationally sampled from the core ventral cervical muscle–adipose region. Previous studies have treated pork jowl as a distinct raw material in pork-quality and authentication research [10], and jowl fat has been shown to exhibit characteristic fatty-acid profiles that vary with production conditions [11,12]. This heterogeneous muscle–adipose architecture may contribute to cut-specific differences in water distribution, water-holding capacity, texture, and lipid composition. In addition, the abundant intramuscular and intermuscular fat may provide lipid substrates for volatile formation during heating. PJ is widely consumed across East Asian culinary systems, serving as the primary raw material for Korean grilled PJ, Cantonese barbecued pork char siu, and Japanese ramen chashu. While fresh PJ is rarely cooked and eaten in most Western countries, Italy produces premium dry-cured guanciale from this cut, resulting in striking regional disparities in its processing and consumption patterns. Chemically, PJ is rich in intramuscular and intermuscular fats, and its fatty-acid composition and volatile flavor generation rules differ markedly from lean pork cuts, endowing it with unique processing potential. Nevertheless, systematic quantitative studies on the eating quality, nutritional value, and flavor profile of PJ remain limited. This knowledge gap constrains evidence-based slaughter grading, precision meat processing, and the development of cut-specific pork products [4]. Regmi et al. [4] recently profiled flavor signatures across indigenous Chinese pig breeds and muscle cuts using multimodal analysis, but did not include PJ. Similarly, Zhang et al. [5] characterized flavor differences among four pork cuts in Chalu black pigs using multi-omics, yet PJ was absent from their sample set.

To address this gap, this exploratory study integrated instrumental quality measurements, proximate and amino/fatty-acid composition, low-field nuclear magnetic resonance (LF-NMR) water distribution, headspace solid-phase microextraction–gas chromatography–mass spectrometry (HS-SPME-GC-MS) volatile profiling, odor activity value (OAV) calculation, and orthogonal partial least squares–discriminant analysis (OPLS-DA) within a matched-animal design. To our knowledge, few studies have applied this combined analytical framework with a specific focus on PJ. The specific objectives were to: (1) provide a preliminary multidimensional characterization of the instrumental quality, nutritional composition, water distribution, and volatile profiles of PJ, pork belly (PB), Boston butt (BB), and spare ribs (SR); (2) identify characteristics that distinguish PJ from the other three commercial cuts; and (3) provide preliminary quantitative information for future cut-specific raw-material selection and product development.

## 2. Materials and Methods

### 2.1. Materials

Ten castrated DLY three-way crossbred finishing pigs with an average slaughter age of 180 ± 3 days and consistent live weight (98.9 ± 5.2 kg) were supplied by Henan Zhongpin Food Co., Ltd. (Xuchang China). All experimental pigs were raised under identical environmental conditions and fed the same commercial compound feed according to standard growth stages.

Before slaughter, all pigs underwent 12 h of lairage with free access to water, followed by 3 h of water deprivation. The pigs were slaughtered following standard industrial procedures, including electrical head-only stunning (70–90 V, 1–3 s), exsanguination, scalding, dehairing, evisceration, and carcass splitting. No electrical stimulation was applied post-slaughter. Carcasses were chilled at 0–4 °C for 24 h in a conventional chilling system before sampling. All slaughter procedures were conducted in accordance with applicable Chinese regulations and animal welfare requirements..

After 24 h of carcass chilling at 0–4 °C, the four pork cuts were collected following the technical specifications of agricultural industry standard NY/T 821-2019 for pork muscle quality determination [13]. Pork jowl (PJ) was obtained from the core ventral cervical muscle–adipose region, pork belly (PB) from the abdominal wall, Boston butt (BB) from the shoulder region, and spare ribs (SR) from the rib/intercostal region. PJ was trimmed of bone fragments, coarse peripheral fascia, and visible tough connective tissue while retaining naturally associated intramuscular and intermuscular fat; external fat was standardized to ≤5 mm. PB was completely de-skinned, the skin-associated subcutaneous fat was removed, and naturally distributed muscle-fat layers were retained after trimming large readily separable fat deposits and coarse fascia. BB was prepared as clean shoulder tissue without scapular, bone, or cartilage residues; coarse fascia and tough connective tissue were removed, external fat was standardized to ≤5 mm, and intramuscular fat was retained. SR was completely deboned, and the intercostal muscles together with their naturally associated intramuscular and intermuscular fat were used; no external subcutaneous fat layer was included. All trimming was performed by the same trained operator using predefined cut-specific criteria.

Approximately 500 g of prepared tissue was collected from each cut of each pig, vacuum-packed, and divided into two subsamples. Samples for nutritional composition, amino-acid, and fatty-acid analyses were stored at −80 °C for no longer than 4 weeks before analysis. Each subsample underwent only one freeze–thaw cycle prior to analysis. Samples for eating quality, LF-NMR, and volatile flavor analyses were analyzed within 48 h post-slaughter from refrigerated (4 °C) storage without freezing.

### 2.2. Eating Quality Analysis

pH was measured at 24 h post-slaughter using a portable pH meter (PHS-3E, Shanghai INESA Scientific Instrument Co., Ltd., Shanghai, China) calibrated with pH 4.01 and 7.00 standard buffers. A pH electrode was inserted directly into the geometric center of each cut to a depth of approximately 2 cm. Ultimate pH (pH_24h_) values are reported. Meat color was measured at 24 h post-slaughter using a chroma meter (CR-400, Konica Minolta, Osaka, Japan) calibrated with a white calibration plate (D65 illuminant, 2° observer angle, 8 mm aperture). Measurements were taken on the freshly cut surface after 30 min of blooming at 4 °C, with three replicate readings per sample.

Cooking loss and centrifugal loss were determined according to the Chinese agricultural standard NY/T 2793-2015 [14]. Meat blocks approximately 2.54 cm thick were weighed before cooking (W_1_), sealed in heat-resistant bags, and heated in a 72 °C water bath until the core temperature reached 70 °C. Core temperature was monitored in real time using a digital probe thermometer (TM-902C, Shanghai Anlesin Instrument Co., Ltd., Shanghai, China) inserted into the geometric center of each sample. Heating time varied among cuts (approximately 25–35 min) depending on tissue composition and sample geometry. The samples were cooled under running water for 30 min, stored at 4 °C for approximately 12 h, blotted dry, and reweighed (W_2_). Cooking loss was calculated as [(W_1_ − W_2_)/W_1_] × 100. For centrifugal loss, approximately 10.0 g of tissue was collected from the center of a 2.0 cm thick meat block and weighed (M_1_). The sample was wrapped in filter paper, placed in a 50 mL centrifuge tube containing defatted absorbent cotton, and centrifuged at 9000 r/min for 10 min at 4 °C. After centrifugation, the filter paper was removed and the sample was reweighed (M_2_). Centrifugal loss was calculated as [(M_1_ − M_2_)/M_1_] × 100.

To standardize mechanical testing among cuts, shear-force and texture profile analysis (TPA) specimens were obtained from representative muscle-rich regions after removing visible fascia and tough connective tissue, while intramuscular fat was retained. For heterogeneous cuts such as PJ and PB, specimens were taken from the largest contiguous muscle region and oriented along the predominant fiber direction. Three specimens per animal and cut were oriented parallel to the muscle fibers and sheared perpendicular to the fibers, and the mean value was recorded. Approximately 100 g of each subsample was heated in a 78 °C water bath. The core temperature of each sample was individually monitored using a digital probe thermometer inserted into its geometric center, and heating was terminated when the core temperature reached 72 °C. Heating time was therefore not fixed and varied slightly among samples depending on cut and sample characteristics. The samples were then cooled to 25 °C. Specimens were standardized by dimensions (1.0 cm × 1.0 cm × 2.0 cm for TPA; 1.0 cm × 1.0 cm × 3.0 cm for shear force).

The shear force was measured according to NY/T 1180-2006 using a TA-XT2i texture analyzer fitted with a Warner–Bratzler shear blade (Stable Micro Systems, Godalming, UK) [15]. The blade/test speed was set at 1 mm/s. TPA was performed using a TA-XT2i texture analyzer (Stable Micro Systems, Godalming, UK) equipped with a 36 mm diameter AACC36 cylindrical probe. Instrument parameters were set as follows: pre-test, test, and post-test speed = 1.0 mm/s; compression = 50% of sample height; trigger force = 5 g; two compression cycles separated by 5 s.

### 2.3. Nutritional Composition Analysis

The proximate composition of the meat samples, including moisture, crude protein, crude fat and ash, was analyzed in accordance with the following Chinese national standard methods: moisture (GB 5009.3-2016) [16], crude protein (GB 5009.5-2016) [17], crude fat (GB 5009.6-2016) [18], and ash (GB 5009.4-2016) [19], respectively. All proximate composition data (moisture, crude protein, crude fat, ash, and carbohydrates) are expressed on a wet-weight (fresh-weight) basis. Moisture was determined by oven drying at 105 °C (DHG-9070A, Shanghai Yiheng Scientific Instrument Co., Ltd., Shanghai, China). Crude protein was determined by the Kjeldahl method using an automatic Kjeldahl nitrogen analyzer (Kjeltec 8400, Foss Analytical AB, Högänas, Sweden). Crude fat was determined by Soxhlet extraction with petroleum ether using a fat extractor (SOX606, Shandong Hanon Instruments Co., Ltd., Jinan, China). Ash was determined by incineration at 550 °C in a muffle furnace (SX2-5-12N, Shanghai Yiheng Scientific Instrument Co., Ltd., Shanghai, China). Carbohydrate content was calculated by difference as carbohydrates (%) = 100 − moisture (%) − crude protein (%) − crude fat (%) − ash (%), and was treated as a calculated residual rather than a directly measured biological component.

### 2.4. Amino-Acid Analysis

Amino-acid composition was determined according to GB 5009.124-2016 [20]. Meat samples were freeze-dried (LGJ-10, Beijing Songyuan Huaxing Technology Development Co., Ltd., Beijing, China) and homogenized to a fine powder. Approximately 0.2 g of freeze-dried sample was hydrolyzed with 6 mol/L HCl at 110 °C for 22 h under nitrogen protection. After cooling, filtration, and constant-volume dilution, the hydrolysate was analyzed using an L-8900 amino-acid analyzer (Hitachi High-Technologies Corporation, Tokyo, Japan) with external standard calibration. Essential amino acids (EAAs) included isoleucine, threonine, valine, methionine, leucine, phenylalanine, lysine, and histidine. Tryptophan was not quantified due to degradation during acid hydrolysis. Histidine was classified as an essential amino acid according to the WHO/FAO/UNU adult amino-acid requirement recommendations [21]. Amino-acid contents were expressed on a wet-weight basis (g/100 g).

### 2.5. Fatty-Acid Analysis

Fatty-acid profiles were analyzed according to GB 5009.168-2016 (Method 3) [22]. Total lipids were extracted using the Folch method [23] (chloroform–methanol, 2:1, *v*/*v*). Fatty acids were transesterified to fatty-acid methyl esters (FAMEs) using methanol–sulfuric acid (10:1, *v*/*v*) at 80 °C for 30 min. FAMEs were analyzed using an Agilent 7890B gas chromatograph (Agilent Technologies, Santa Clara, CA, USA) equipped with a flame ionization detector (FID) and a SP-2560 capillary column (100 m × 0.25 mm i.d., 0.20 μm film thickness; Supelco, Bellefonte, PA, USA). The injector and detector temperatures were 250 °C and 260 °C, respectively. The oven temperature program was: initial 140 °C (5 min), ramp to 240 °C at 4 °C/min (hold 15 min). Nitrogen served as the carrier gas. FAMEs were identified by comparison with retention times of a 37-component FAME standard mixture (Supelco 37 Component FAME Mix, Sigma-Aldrich, St. Louis, MO, USA). Fatty acids were reported as relative percentages of total identified fatty acids. The polyunsaturated-to-saturated fatty-acid ratio (PUFA/SFA) and the n-6-to-n-3 polyunsaturated fatty-acid ratio (n-6/n-3 PUFA) were calculated for each biological replicate. The atherogenic index (AI) and thrombogenic index (TI) were calculated according to Covaciu et al. [24].

(1) $$AI=\frac{C12:0+4\times C14:0+C16:0}{\Sigma MUFA+\Sigma n\text{-}6\;PUFA+\Sigma n\text{-}3\;PUFA}$$

(2) $$TI=\frac{C14:0+C16:0+C18:0}{0.5\times \Sigma MUFA+0.5\times \sum n\text{-}6\;PUFA+3\times \Sigma n\text{-}3\;PUFA+\frac{\Sigma n\text{-}3\;PUFA}{\Sigma n\text{-}6\;PUFA}}$$

where C12:0, C14:0, C16:0, and C18:0 represent lauric, myristic, palmitic, and stearic acids, respectively. MUFA denotes monounsaturated fatty acids, and PUFA denotes polyunsaturated fatty acids; ∑n-3 PUFA and ∑n-6 PUFA refer to the total contents of n-3 and n-6 polyunsaturated fatty acids, respectively.

### 2.6. Low-Field Nuclear Magnetic Resonance (LF-NMR) Measurement

LF-NMR measurements followed Yang et al. [25] with modifications to sample mass, tube size, measurement temperature, and CPMG acquisition parameters, as specified below. Approximately 2.0 g of raw tissue was excised as a single intact piece from the central edible portion of each cut. For PJ and PB, the sampled piece retained the naturally associated muscle and intramuscular/intermuscular fat; no multi-site pooling or homogenization was performed. Visible fascia, tough connective tissue, and external subcutaneous fat were removed, while intramuscular and intermuscular fat were retained. Samples were wrapped in PTFE tape and placed in 25 mm NMR tubes. The results therefore represent the prepared central edible fraction rather than the intact commercial cut.

Transverse relaxation time (T_2_) spectra were acquired on a MesoMR23-060H-I low-field NMR analyzer (Suzhou Niumag Analytical Instrument Co., Ltd., Suzhou, China) equipped with a 0.5 T magnet (proton resonance frequency = 21.24 MHz) stabilized at 32 °C. The Carr–Purcell–Meiboom–Gill (CPMG) pulse sequence was adopted with the following parameters: repetition wait time (TW) = 2000 ms; echo number = 10,000; echo time (TE) = 0.1 ms; number of scans = 4; 90° pulse width ($P_{1}$) = 5.5 μs; 180° pulse width ($P_{2}$) = 12.0 μs. Multi-exponential inversion of T_2_ relaxation decay curves was completed via the NMR Analysis software package (Ver 4.0, Suzhou Niumag Analytical Instrument Co., Ltd., Suzhou, China) based on the simultaneous iterative reconstruction technique (SIRT) algorithm to generate T_2_ relaxation spectra.

### 2.7. Volatile Flavor Analysis

Volatile flavor compounds were characterized by HS-SPME-GC-MS using a matched subset of six individual pigs randomly selected from the ten animals included in the study. All four cuts (PJ, PB, BB, and SR) from each selected pig were analyzed, providing six biological replicates per cut (*n* = 6; 24 samples in total). Because HS-SPME-GC-MS analysis is relatively time- and instrument-intensive, this six-pig subset was used to maintain a balanced within-animal comparison among the four cuts while keeping the analytical workload feasible. Briefly, 4 g of homogenized pork tissue was transferred into a 20 mL headspace vial, mixed with 5 mL of 25% (*w*/*v*) NaCl solution and 10 μL of 500 ppm internal standard (2-methyl-3-heptanone, 99%, Sigma-Aldrich, St. Louis, MO, USA). The vial was tightly sealed and equilibrated in a water bath at 80 °C for 15 min. A 50/30 μm divinylbenzene/carboxen/polydimethylsiloxane (DVB/CAR/PDMS) SPME fiber (Supelco, Bellefonte, PA, USA) was exposed to the vial headspace at 80 °C for 50 min to extract volatile analytes. After extraction, the fiber was inserted into the GC inlet for thermal desorption at 250 °C for 5 min [26]. The 80 °C heating step was designed to simulate the early cooking process of pork, which could release thermally unstable volatile substances and accelerate the formation of volatile products originating from lipid oxidation.

GC separation was performed on an Agilent 7890A gas chromatograph (Agilent Technologies, Santa Clara, CA, USA) fitted with an HP-5 MS capillary column (30 m × 0.25 mm i.d., 0.25 μm film thickness; Agilent Technologies, Santa Clara, CA, USA). High-purity helium (≥99.999%) served as the carrier gas at a constant flow rate of 1.0 mL/min under splitless injection mode. The injector temperature was maintained at 250 °C, and the oven temperature program was set as follows: initial temperature of 40 °C with a 5 min hold time, ramped to 180 °C at 3 °C/min and held for 2 min, then raised to 230 °C at 10 °C/min with a 3 min holding period.

Mass spectrometric detection was conducted using an Agilent 5975C mass spectrometer (Agilent Technologies, Santa Clara, CA, USA) with electron ionization (EI) at 70 eV. The ion source temperature was set to 230 °C, and full-scan mass spectra were acquired across an *m*/*z* range of 35–500. Chromatographic signals were processed using MassHunter Qualitative Analysis software (Version 12.0, Agilent Technologies, Santa Clara, CA, USA). A chromatographic signal was considered detected when its signal-to-noise ratio was ≥3 and the corresponding peak could be reliably integrated. For a compound tentatively identified elsewhere in the dataset, ND was assigned when the corresponding signal in a sample had an S/N ratio < 3 or could not be reliably integrated. Peaks meeting the detection criterion were compared with the NIST23 mass spectral library, and compounds with a library similarity score of ≥80% were tentatively identified [26].

The concentration of each volatile compound (Ci, μg/kg) was semi-quantified using an internal-standard method, with the relative response factor (RRF) assumed to be 1.0. Because the RRF was assumed to be 1.0 for all compounds, the reported concentrations are semi-quantitative estimates. The results are suitable for relative comparison among cuts but not for quantitative comparison with studies using compound-specific calibration. The calculation formula was as follows:

(3) $$C_{i}=\frac{A_{i}}{A_{IS}}\times \frac{C_{IS}\times 10^{-3}\times V_{IS}}{m}\times 1000$$

where A_i_ = peak area of the target volatile compound, A_IS_ = peak area of the internal standard (2-methyl-3-heptanone), C_IS_ = concentration of the internal standard solution (500 μg/mL), V_IS_ = volume of the internal standard added (10 μL), and m = mass of pork sample (4 g). The factor of 1000 was used for unit conversion from μg/g to μg/kg.

Nominal odor activity values (OAVs) were calculated using the semi-quantified abundance of each compound and its published odor threshold [27]:

(4) $$OAV=\frac{Ci}{Ti}$$

where $C_{i}$ represents the concentration of the target compound in pork (μg/kg), and $T_{i}$ refers to its odor threshold (μg/kg), cited from aqueous matrix data in *Odour Thresholds: Compilations of Odour Threshold Values in Air, Water and Other Media* (2nd enlarged and revised edition) [27]. It should be noted that odor thresholds measured in different matrices are not directly interchangeable with thresholds in pork muscle tissue, which represents an inherent limitation of the OAV calculation approach in this study. Compounds with nominal OAV > 1 were considered potentially aroma-relevant under the assumptions used in the present calculation. Cumulative OAV was defined as the sum of individual nominal OAVs for all compounds with nominal OAV > 1 in each cut, providing a comparative index of overall potential aroma relevance.

### 2.8. Statistical Analysis

All raw experimental datasets were collated and subjected to preliminary calculations in Microsoft Excel 2016. Data were analyzed using IBM SPSS Statistics 26.0 (IBM Corp., Armonk, NY, USA). Technical replicate measurements were averaged within each pig and cut before statistical analysis. Individual pigs were the biological experimental unit. Since each pig supplied four cuts, data were analyzed using linear mixed-effects models with cut as a fixed effect and pig as a random intercept. Diagonal, compound-symmetry, and unstructured covariance structures were compared for the repeated measurements within each pig, and the unstructured structure was selected based on the lowest AIC and BIC values. The repeated covariance structure accounted for residual correlations among the four cuts within the same pig. Model assumptions were evaluated using Q–Q plots and the Shapiro–Wilk test for residual normality, and plots of standardized residuals versus fitted values for homoscedasticity. No variable transformation was applied. When the cut effect was significant (*p* < 0.05), pairwise comparisons were performed using a Tukey-adjusted estimated marginal means test. The same mixed model was applied to volatile compounds. Benjamini–Hochberg FDR correction was applied across the overall cut-effect *p*-values of the individual volatile-compound models. For compounds with q < 0.05, pairwise comparisons of estimated marginal means were performed using Tukey adjustment. Results were expressed as mean ± SD.

OriginPro 2021 (OriginLab Corporation, Northampton, MA, USA) was employed for conventional figure plotting. Venn diagrams and partial correlation heatmaps were generated via the online tool Chiplot (https://chiplot.online, accessed on 8 April 2026). Orthogonal partial least squares–discriminant analysis (OPLS-DA), a multivariate chemometric approach, was carried out using SIMCA 14.1 (MKS Umetrics, Umeå, Sweden). For OPLS-DA, compounds with ≥50% ND values were excluded; remaining ND values were treated as missing data. The data were unit variance (UV) scaled without prior transformation. The model included 24 observations from six pigs, and validation used six-fold leave-one-pig-out cross-validation, 200 permutation tests, and CV-ANOVA. Benjamini–Hochberg FDR-adjusted *p*-values are hereafter reported as q values. VIP > 1 and q < 0.05 were predefined as the statistical criteria for candidate discriminant variables; OAV > 1 was used as an additional aroma-relevance screen. For partial correlation analysis, pig identity was treated as a categorical control factor, and correlations were calculated between variables after removing pig-specific effects. All *p*-values were adjusted using the Benjamini–Hochberg FDR procedure. Instrumental quality, proximate composition, amino-acid composition, fatty-acid composition, and LF-NMR analyses were based on all ten pigs (*n* = 10 per cut), whereas volatile-compound, OAV, and OPLS-DA analyses were based on the matched six-pig subset (*n* = 6 per cut). Partial correlation analysis used matched nutritional and volatile data from these same six pigs.

## 3. Results and Discussion

### 3.1. Analysis of Instrumental Quality

Significant differences (*p* < 0.05) in cooking loss and centrifugal loss were observed among the four pork cuts (Table 1). These parameters were used as indirect indicators of the water-retention ability of the meat samples. PJ exhibited the highest cooking loss (26.35%) and centrifugal loss (28.07%), while PB and SR had the lowest cooking loss, and BB had comparable centrifugal loss to PB and SR; all three were significantly lower than PJ, which may partly reflect differences in tissue architecture and composition among cuts. The cooking loss of PB in the present study (18.39%) was close to the values reported by Hoa et al. [28] for pork belly (17.29–17.50%), whereas the cooking loss of BB (20.19%) was somewhat lower than that reported for shoulder butt (24.40–25.33%). Hoa et al. [28] also reported pH values of approximately 5.79–5.92 for pork belly and shoulder butt, broadly comparable with the pH24h range observed here (5.70–5.91). These comparisons suggest that water-holding capacity and ultimate pH vary not only among cuts but also with sample composition and experimental conditions.

Instrumental color differed among cuts. PJ had the highest L* (64.53) values and the lowest a* (1.04); BB showed the lowest L* (35.18) and b* (9.54) values with a high a* (6.02); PB had the highest a* (7.62), and SR showed the numerically highest b* value (16.79). The color values differed more substantially from those reported by Hoa et al. [28], particularly for L and a, which may reflect differences in anatomical composition, trimming procedures, production background, and the specific tissue area selected for color measurement.

Shear force and TPA parameters confirmed textural divergence. PJ had the highest shear force (2406.00 gf), hardness (3974.25 gf), and chewiness (10,354.33), indicating the toughest texture. PB showed the lowest shear force (1771.67 gf), hardness (2333.00 gf), and chewiness (5602.33), exhibiting the most tender texture. Springiness was lowest in BB (3.71), while resilience was lowest in SR (0.26). The shear force values of PJ observed in this study (approximately 23.6 N) are lower than the reported shear forces of 38.89–47.25 N for the longissimus thoracis by Xu et al. [29]. These differences may reflect variation in anatomical origin, muscle–fat architecture, sample preparation, and shear-force instrumentation. Previous studies have also demonstrated marked muscle-dependent variation in pork texture. Li et al. [30] reported differences in shear force and TPA parameters between pork muscles and showed that collagen characteristics were associated with hardness, cohesiveness, chewiness, and resilience. Thus, the higher hardness and chewiness of PJ observed here may partly reflect its distinct muscle–connective tissue architecture, although collagen characteristics were not directly measured in the present study.

Overall, PJ exhibited lower instrumental tenderness and water-retention ability than the other cuts under the present experimental conditions. Whether these characteristics can be improved by specific processing treatments requires further investigation.

### 3.2. Analysis of Nutritional Composition

Nutritional profiles of four pork cuts were summarized in Table 2. Crude protein and fat, key pork nutrients, differed significantly (*p* < 0.05) across cuts. BB showed the numerically highest protein (18.10%) and the lowest fat (13.03%), presenting a distinct high-protein and low-fat profile. In contrast, PB and SR exhibited higher fat contents and lower protein contents. Their higher fat contents would be expected to increase energy density, although energy content was not directly determined. Moisture followed the sequence: BB > PJ > PB ≈ SR. Calculated carbohydrate and ash contents showed no significant differences among cuts (*p* > 0.05).

The compositional differences among cuts are consistent with their anatomical characteristics. BB contained a greater proportion of muscle tissue and less extractable fat, whereas PJ and SR are muscle–adipose complexes containing more naturally associated intermuscular fat. A similar cut-related pattern was reported by Hoa et al. [28], who found higher fat and lower moisture contents in pork belly than in shoulder butt. This agreement supports an important contribution of anatomical location and tissue composition to the observed differences. Because all four cuts in the present study were obtained from the same DLY pigs under identical production conditions, within-study differences are less likely to reflect breed or production-system effects. In contrast, absolute values differed from those reported for free-range Mangalica pork by Nikolic et al. [31], which may reflect differences in breed, production system, slaughter characteristics, and sampling or trimming procedures.

The carbohydrate content calculated by difference (<0.6% for all cuts) is negligible and primarily reflects accumulated analytical uncertainty rather than physiologically meaningful carbohydrate content.

### 3.3. Analysis of Amino-Acid Composition

Amino-acid profiles of four pork cuts are listed in Table 3. Total amino acids (TAA), essential amino acids (EAAs), and individual amino acid contents differed significantly (*p* < 0.05) among samples. BB accumulated the highest TAA (17.58 g/100 g), followed closely by PJ (17.26 g/100 g) and PB (17.09 g/100 g); these three did not differ from each other, but all exceeded SR (13.99 g/100 g, *p* < 0.05).

For EAAs, BB contained the highest level (7.67 g/100 g) and SR the lowest (6.16 g/100 g). The EAA/TAA ratio, which reflects the proportion of measured essential amino acids in total amino acids, ranged narrowly from 0.42 to 0.44 among the four cuts. Although the ratio was statistically lower in PJ (0.42) than in the other cuts (0.43–0.44, *p* < 0.05), the absolute difference was small (≤0.02), indicating broadly similar EAA proportions. BB also exhibited significantly greater lysine (1.79 g/100 g) than other parts; lysine was the most abundant quantified EAA in all four cuts, ranging from 1.44 to 1.79 g/100 g. The methionine content of PB (0.15 g/100 g) was approximately twice that of PJ and BB (0.07 g/100 g each). However, because methionine quantification can be sensitive to acid-hydrolysis conditions, this relatively large difference should be interpreted cautiously. Collectively, BB provided the highest absolute TAA and EAA contents among the four prepared cuts. For anatomically comparable commercial pork cuts, Fanelli et al. [32] reported lysine, leucine, and glutamic acid contents of 2.04, 1.85, and 3.36 g/100 g, respectively, in shoulder butt, and 1.85, 1.67, and 2.97 g/100 g in back ribs. These values were somewhat higher than those observed for BB (1.79, 1.50, and 3.06 g/100 g) and SR (1.44, 1.19, and 2.48 g/100 g), respectively, in the present study. Such differences may reflect variation in genotype, tissue composition, trimming procedures, and analytical conditions. SR showed lower absolute contents of TAA, EAAs, NEAAs, and most individual amino acids. However, its EAA/TAA and EAA/NEAA ratios were comparable to those of PB and BB, indicating that the lower amino-acid content was mainly associated with lower total protein content rather than poorer amino-acid balance.

### 3.4. Analysis of Fatty-Acid Profile

The fatty-acid composition of the four pork cuts is presented in Table 4. The results of the present study demonstrated that C16:0, C18:0, C18:1n9c and C18:2n6 were the predominant fatty acids in meat from DLY pigs, with their contents all exceeding those of other fatty acids; this finding is consistent with a previous study [24,29]. The overall fatty-acid pattern was broadly consistent with previous pork studies. Covaciu et al. [24] reported SFA, MUFA, and PUFA contents of 44.57–50.70%, 31.09–40.53%, and 14.92–18.20%, respectively, across pork leg, loin, and tenderloin, whereas the corresponding ranges in the present study were 40.43–44.66%, 43.19–49.92%, and 9.69–12.19%. Xu et al. [29] reported C16:0 (24.47–26.15%), C18:0 (14.02–14.99%), C18:1n9c (38.18–43.84%), and C18:2n6c (7.99–12.17%) in DLY longissimus muscle, broadly comparable with the ranges observed here. Differences in absolute values may reflect anatomical location, tissue composition, slaughter weight, and dietary conditions.

PJ had the lowest total saturated fatty acid (SFA) content (40.43%), markedly lower than BB (43.56%), PB (43.18%) and SR (44.66%), while these three cuts showed comparable SFA levels. Stearic acid (C18:0) drove this divergence: PJ had less C18:0 (12.17%) than other cuts (14.67–15.80%), whereas C14:0 and C16:0 were unchanged (*p* > 0.05). This pattern may reflect cut-specific lipid deposition and fatty-acid metabolism, but the underlying enzymatic mechanisms require further validation [33].

Conversely, PJ possessed the highest MUFAs (49.92%, *p* < 0.05), exceeding BB (44.83%), PB (45.44%) and SR (43.19%). Elevated palmitoleic acid (C16:1, 3.95%) accounted for this gap, and PJ had significantly higher oleic acid (C18:1, 44.93%) than PB, BB, and SR. For PUFAs, SR ranked first (12.19%), followed by BB (11.77%) and PB (11.38%), with PJ the lowest (9.69%). The higher PUFA content of SR (12.19%) may also increase its susceptibility to lipid oxidation during storage and cooking, which is consistent with the higher total volatile compound content observed in this cut (Section 3.6). Total n-3 PUFAs differed significantly among cuts, with PB, BB, and SR showing higher values than PJ. PB and SR showed the highest DHA levels (*p* < 0.05). The n-6/n-3 PUFA ratio was highest in PJ (16.81) and lower in PB, BB, and SR (9.27–11.07). These ratios describe the relative fatty-acid composition of each cut and should not be interpreted as direct measures of the nutritional adequacy of the overall diet.

AI and TI are fatty-acid-based indices used to describe the relative atherogenic and thrombogenic potential of lipid profiles, with lower values generally interpreted more favorably from a lipid nutritional perspective [34,35]. PJ showed the lowest numerical AI and TI values among the four cuts. Lower AI and TI values are generally interpreted as reflecting a lower relative contribution of the fatty acids included in the numerators of these indices. However, these indices do not account for the total amount of fat consumed.

### 3.5. Analysis of LF-NMR Water Distribution

**Figure 1:**
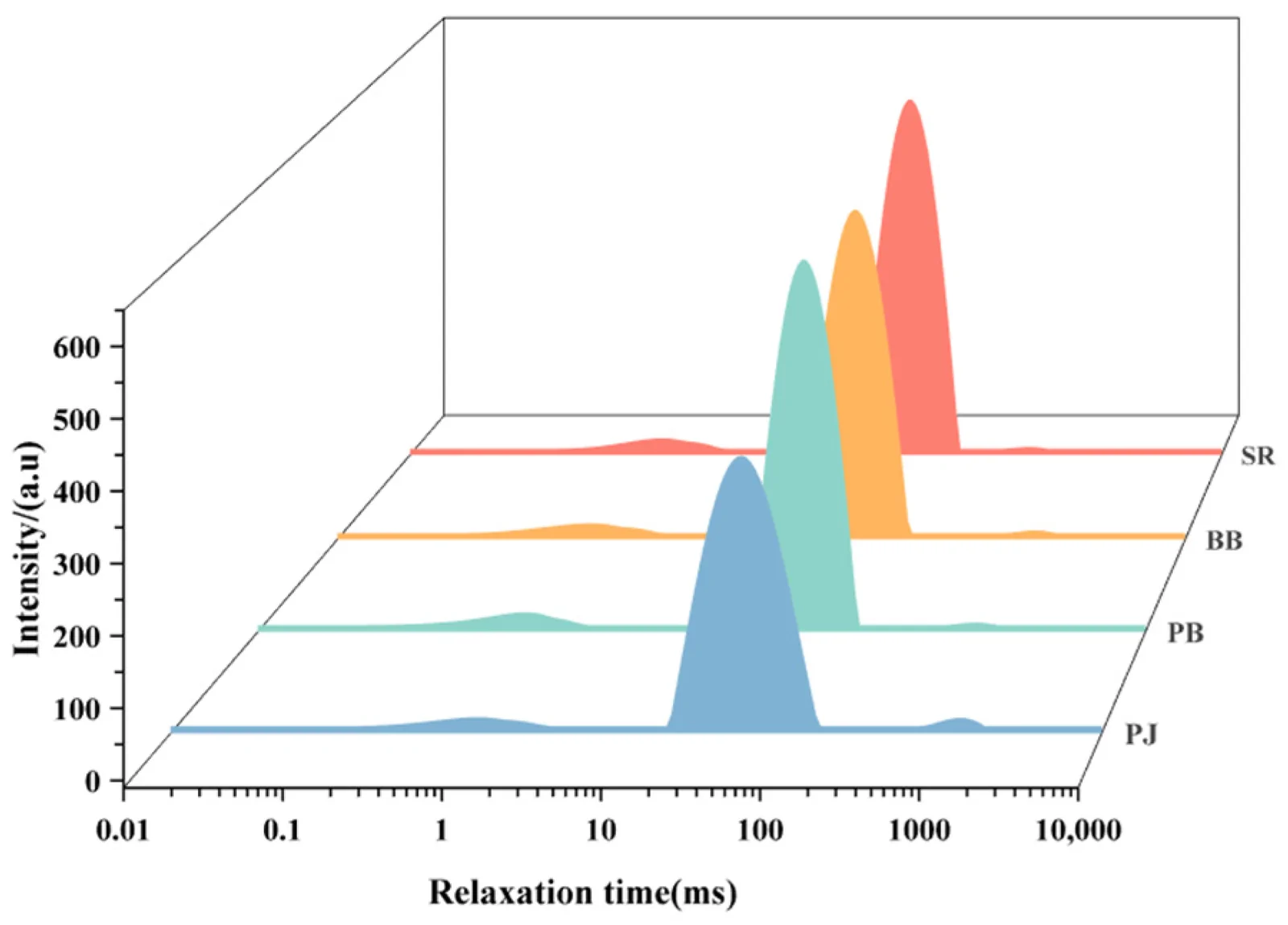
LF-NMR T_2_ relaxation spectra of different pork cuts. The three peaks correspond to T_21_ (bound water, 0–10 ms), T_22_ (immobilized water, 10–100 ms), and T_23_ (free water, >100 ms) populations discussed in the text.

T_2_ relaxation spectra (Figure 1) revealed three water populations: T_21_ (0–10 ms, bound water associated with myofibrillar proteins), T_22_ (10–100 ms, immobilized water within myofibrils), and T_23_ (>100 ms, free water in extracellular spaces) [36]. Relaxation times and peak area proportions are summarized in Table 5.

Significant differences in T_2_ were observed across cuts (*p* < 0.05). SR exhibited the shortest T_21_ (0.69 ms), followed by PB (0.74 ms) and PJ (0.97 ms), while BB showed the longest T_21_ (1.43 ms). No significant differences in T_22_ were detected among the four cuts. Because T_22_ is generally associated with immobilized water within the myofibrillar structure [37], the present results suggest that this water fraction showed relatively limited variation among cuts. T_23_ followed the order PJ > PB ≈ BB > SR, indicating that the free water fraction in PJ had greater mobility [37,38].

Regarding peak area proportions, PB possessed the maximum P21 (3.99%) and PJ the minimum (3.49%). No inter-group differences were detected for P22 (*p* > 0.05). Notably, PJ had a significantly higher P23 (1.08%) than other cuts (*p* < 0.05). These peak-area proportions were broadly comparable with those reported by Xu et al. [29] for the longissimus thoracis of Duroc × Landrace × Yorkshire pigs. Bertram et al. [37] also reported significant relationships between LF-NMR transverse relaxation characteristics and pork water-holding capacity measured by centrifugation and drip-loss methods. Consistent with this relationship, the higher T_23_ and P23 values of PJ in the present study occurred concurrently with its greater cooking and centrifugal losses, supporting an association between a larger least-restricted water fraction and reduced water retention. However, direct numerical comparison with Bertram et al. [37] is limited by differences in NMR component fitting and muscle/sample characteristics. The elevated P23 of PJ may therefore be consistent with its higher cooking loss (26.35%) and centrifugal loss (28.07%) compared to the other cuts. Because subcutaneous fat was removed and only the prepared central edible fraction was analyzed, the LF-NMR comparisons apply to the standardized tissue fraction rather than the intact commercial cuts, particularly for cuts that differ substantially in external fat deposition (e.g., PJ and PB).

### 3.6. Analysis of Volatile Flavor Compounds

**Figure 2:**
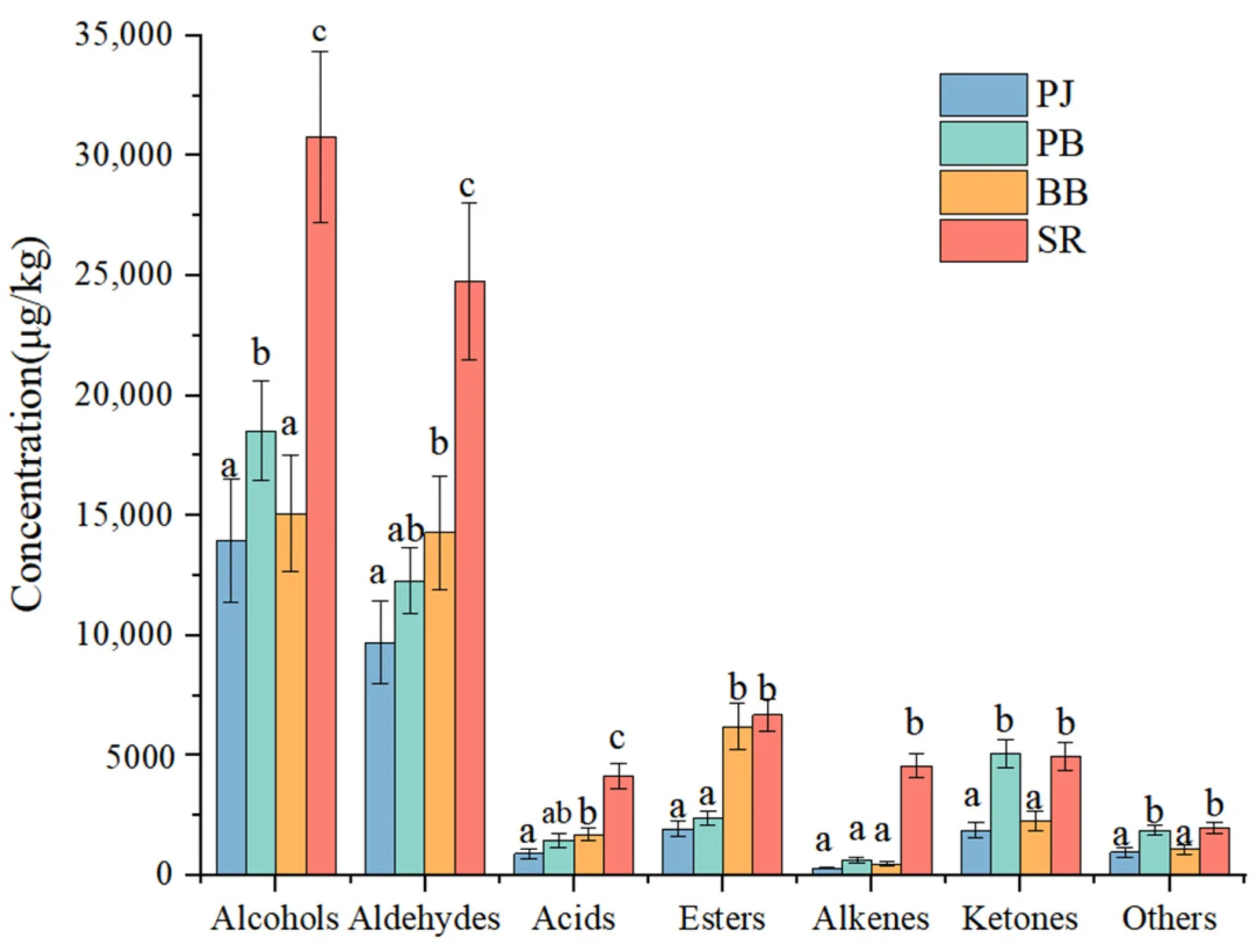
Total contents of volatile compound classes of different pork cuts. Values represent the sum of semi-quantified concentrations (μg/kg, assuming RRF = 1.0) within each chemical class. Different lowercase letters indicate significant differences among cuts within the same chemical class (*p* < 0.05).

Sixty-five compounds across seven chemical classes were tentatively identified in the headspace generated during the standardized 80 °C HS-SPME procedure (Table 6): 28 alcohols, 12 aldehydes, 8 acids, 9 esters, 4 alkenes, 1 ketone, and 3 other compounds. Figure 2 presents the total contents of each volatile compound class in the four pork cuts, while the overall total volatile content of all compounds followed the order: SR (77,699.08 µg/kg) > PB (42,259.42 µg/kg) > BB (41,149.03 µg/kg) > PJ (29,667.14 µg/kg). Marked cut-dependent differences in volatile profiles have also been reported in previous pork studies. Chen et al. [3] found differences in the total volatile flavor compound content among longissimus dorsi, rib muscle, and tendon meat of Duroc–Bamei pigs, with longissimus dorsi showing the highest total volatile abundance. Similarly, Zhang et al. [5] demonstrated distinct VOC profiles among longissimus thoracis, trapezius, hamstring, and belly cuts of Chalu black pigs. These findings are consistent with the pronounced cut-related variation observed in the present study, although direct quantitative comparison is limited by differences in genotype, anatomical sampling, and analytical methodology.

Alcohols, which can be generated from lipid-oxidation-derived aldehydes through reduction and from other enzymatic or thermal reactions, dominated quantitatively [39]. SR accumulated the highest total alcohols (30,778.57 µg/kg), approximately 2.2-fold higher than PJ (13,950.44 µg/kg). 1-Octen-3-ol, a key contributor to pork aroma with a low odor threshold (7 µg/kg), reached 5277.83 µg/kg in SR (17.1% of total alcohols in this cut). This compound derives from linoleic and arachidonic acid oxidation via the 5-lipoxygenase pathway [40]; its elevated concentration in SR is consistent with the higher PUFA content of this cut (12.19%).

Aldehydes, as primary products of unsaturated fatty-acid oxidation, have low odor thresholds and contribute significantly to meat flavor [41]. Total aldehyde content followed SR (24,757.15 µg/kg) > BB (14,283.88 µg/kg) > PB (12,283.88 µg/kg) > PJ (9724.42 µg/kg). Hexanal peaked in SR (17,314.58 µg/kg), whereas nonanal (fatty aroma) was highest in PB (771.74 µg/kg). Hoa et al. [28] also identified hexanal as an important variable aldehyde in pork belly and nonanal as a differentiating aldehyde in shoulder butt.

Esters, formed by alcohol–acid esterification, impart fruity and floral notes [3,42]. Total ester content was highest in SR (6691.50 µg/kg) and BB (6214.02 µg/kg). Ketones, secondary lipid oxidation products, were enriched in SR (4963.33 µg/kg) and PB (5085.39 µg/kg). Alkenes were markedly enriched in SR (4556.43 µg/kg).

**Figure 3:**
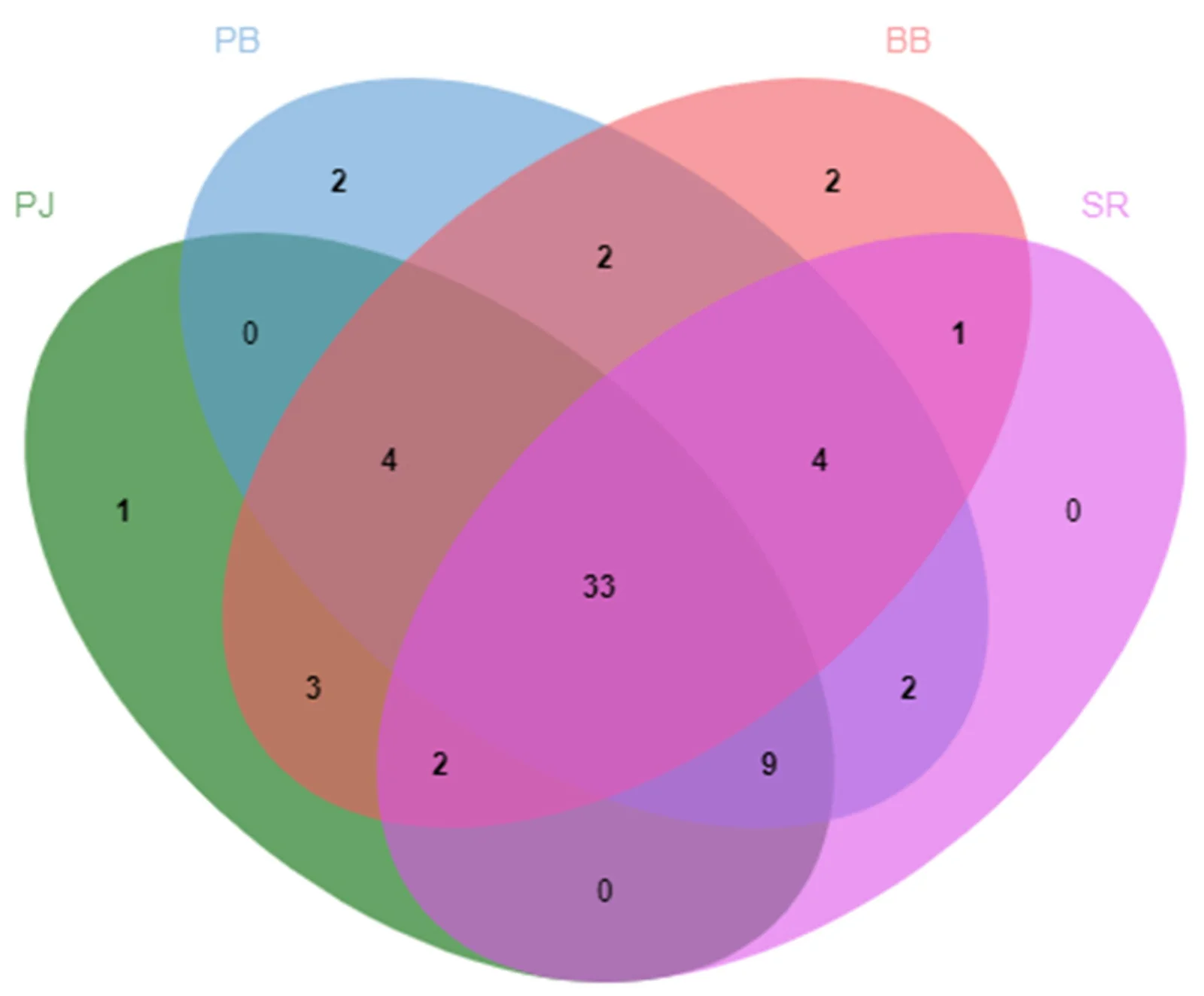
Venn diagram of volatile compounds identified in different pork cuts. Numbers indicate the number of volatile compounds unique to or shared among the corresponding cuts.

Venn diagram analysis (Figure 3) identified 33 compounds common to all four cuts (50.77% of total detected compounds), representing the volatile compounds common to all four cuts. BB contained two compounds not detected in the other cuts: cyclobutanol and 2-methyl-octanoic acid.

While the above analysis describes the quantitative distribution of volatile compounds among cuts, compound abundance alone does not determine aroma contribution. The sensory relevance of each compound depends on its odor threshold, which varies by several orders of magnitude across chemical classes. In the following section, OAVs are calculated to evaluate the potential aroma relevance of each compound and to identify potentially aroma-relevant compounds differentiating the four cuts.

### 3.7. Nominal OAV Analysis

Compounds with nominal OAV > 1 were considered potentially aroma-relevant under the assumptions of the present OAV calculation [43]. Of the 65 identified volatiles, 30 compounds exceeded this threshold across the four cuts (Table 7), predominantly alcohols, aldehydes, esters, ketones, and other compounds.

Alcohols and aldehydes accounted for many of the compounds with relatively high nominal OAVs. 1-Octen-3-ol, a typical mushroom and earthy odorant [44], exhibited its highest OAV in SR (753.98). (E)-3-Hexen-1-ol and 1-dodecanol were associated with green and waxy notes in PB (OAV 61.98 and 222.12, respectively). 2-Butyl-1-octanol showed a high nominal OAV in BB and has been associated with floral/fruity odor descriptors in published sources [43]. Among aldehydes, heptanal exhibited an universally high OAV across all samples. (E)-2-Nonenal showed its highest nominal OAV (2372.60) in SR and may therefore have relatively high potential aroma relevance in this cut. Nonanal achieved its maximum OAV in PB (771.74). Hexanal exhibited moderate OAVs in all experimental groups, which was potentially relevant to the baseline green and apple-like characteristic aroma of pork [45]. Esters, ketones and 2-pentylfuran further differentiated the flavor of different cuts. Methyl decanoate and methyl nonanoate were associated with rich fruity notes in BB, while 2,3-octanedione was potentially relevant to the buttery and fatty aromatic notes in PB and SR. 2-Pentylfuran with fruit and vegetable aroma was a common characteristic component in all samples [46].

Overall, SR showed the highest nominal cumulative OAV and the largest number of high-OAV compounds, followed by PB and BB, whereas PJ presented the minimum cumulative OAV. However, cumulative OAV did not necessarily vary in proportion to total volatile abundance because individual compounds differed markedly in their odor thresholds. For example, 1-decanol was present at relatively low concentrations (13.47–56.93 μg/kg) but exhibited OAVs of 19.24–81.32 because of its low odor threshold (0.7 μg/kg). In contrast, 2-ethyl-1-hexanol occurred at substantially higher concentrations (1093.41–2210.16 μg/kg), but its higher odor threshold (300 μg/kg) resulted in lower OAVs (3.64–7.37). These examples demonstrate that compound abundance alone does not directly indicate potential aroma relevance.

Nonetheless, OAV calculation relies on odor thresholds measured in simple aqueous/organic solvent systems, which cannot fully simulate the complex meat matrix environment. Volatile compound interactions, protein–lipid binding, and human sensory perception differences are neglected in this model. Furthermore, OAVs are not necessarily additive and do not account for synergistic or masking effects, matrix binding, or perceptual interactions. Therefore, follow-up research should combine gas chromatography–olfactometry (GC-O) and aroma recombination sensory tests to further validate the sensory relevance of the identified candidate aroma-relevant compounds.

### 3.8. Orthogonal Partial Least Squares–Discriminant Analysis (OPLS-DA) of Volatile Compounds

**Figure 4:**
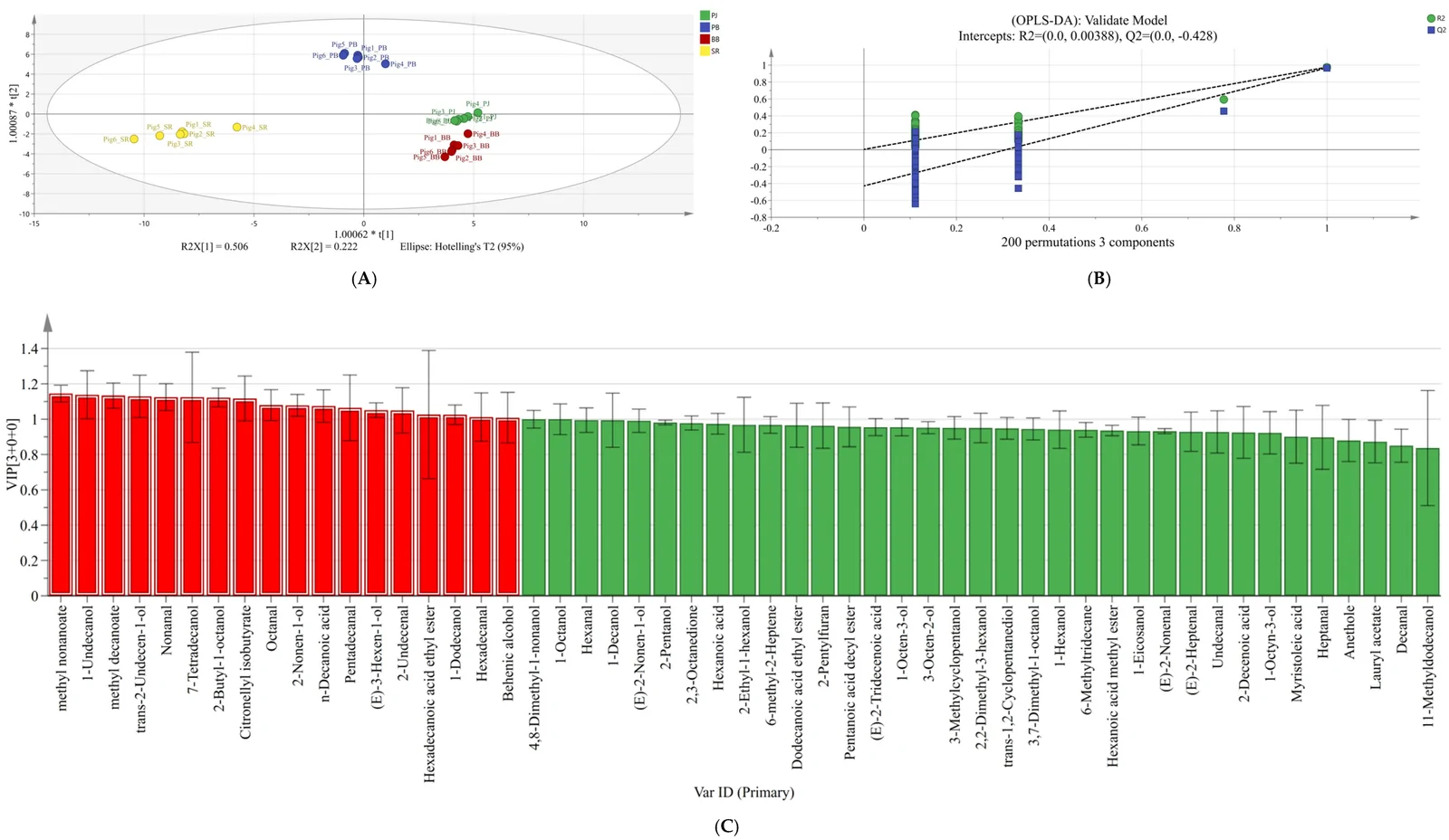
OPLS-DA analysis based on volatile compounds of four pork cuts. (**A**) Score plot; (**B**) permutation validation; (**C**) VIP score ranking. In panel (**C**), red bars indicate variables with VIP > 1, whereas green bars indicate variables with VIP ≤ 1. The asterisk (*) denotes multiplication in the axis notation.

To further screen differential aroma markers that distinguish flavor fingerprints of the four pork cuts, OPLS-DA was subsequently conducted based on all quantified volatile compounds (Figure 4) [47,48]. After excluding 12 volatile compounds with ≥50% non-detected values, 53 compounds were retained, and the remaining non-detected values were treated as missing data. The OPLS-DA model contained three predictive components and no orthogonal component (3 + 0 + 0), with R^2^X(cum), R^2^Y(cum), and Q^2^(cum) values of 0.923, 0.978, and 0.972, respectively (Figure 4A). Model validation was performed using six-fold leave-one-pig-out cross-validation, ensuring that all four samples from the same pig were assigned to the same validation fold. The 200-permutation test (Figure 4B) yielded R^2^ and Q^2^ intercepts of 0.0039 and −0.428, respectively, and CV-ANOVA confirmed model significance (F = 101.559, df = 18, *p* = 0.00015). These results indicate distinct volatile flavor profiles among the four anatomical sites, although the findings should be interpreted as exploratory because only six independent pigs were included.

Eighteen volatile compounds met the combined criteria of VIP > 1 and q < 0.05 (Figure 4C). Applying the additional criterion of OAV > 1 narrowed this set to 10 candidate discriminant markers associated with flavor differentiation: (E)-3-hexen-1-ol, 2-nonen-1-ol, 1-undecanol, 2-butyl-1-octanol, 1-dodecanol, octanal, nonanal, methyl nonanoate, citronellyl isobutyrate and methyl decanoate. These markers require validation in an independent sample set before they can be considered reliable indicators.

### 3.9. Correlations Between Candidate Aroma-Relevant Compounds and Nutritional Components

**Figure 5:**
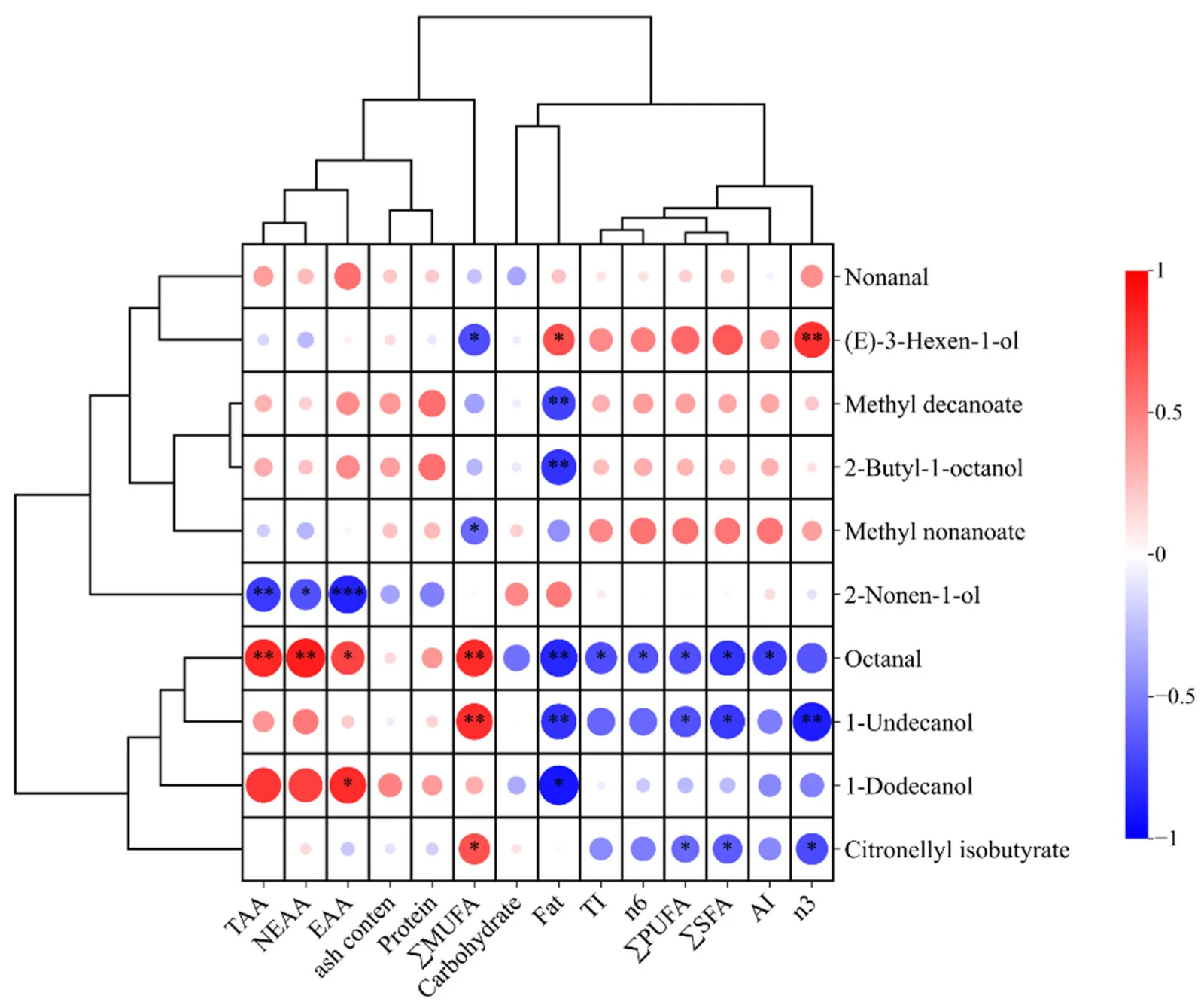
Partial correlation heatmap between candidate aroma-relevant compounds and nutritional variables after adjustment for pig identity. Note: The color gradient represents partial correlation coefficients. Red indicates positive correlation; blue indicates negative correlation. Significance markers denote FDR-corrected thresholds: * *p* < 0.05, ** *p* < 0.01, *** *p* < 0.001.

Partial correlation analysis was performed to examine the relationships between candidate aroma-relevant compounds and nutritional composition indices of samples, visualized via a correlation clustering heatmap (Figure 5). Hierarchical clustering was simultaneously applied to both volatile compounds and nutritional parameters to classify variables with similar correlation patterns. The analysis was based on 24 individual observations (4 cuts × 6 pigs) matched for both volatile and nutritional datasets.

Distinct correlation trends were observed between different categories of volatiles and nutrients. Octanal was positively associated with TAA, NEAAs, EAAs, and MUFAs and negatively associated with fat, TI, n-6 PUFAs, total PUFAs, total SFAs, and AI. 1-Undecanol was positively associated with MUFAs and negatively associated with fat, total PUFAs, total SFAs, and n-3 PUFAs. By contrast, (E)-3-hexen-1-ol was negatively associated with MUFAs and positively associated with fat and n-3 PUFAs. 2-Nonen-1-ol was negatively associated with TAA, NEAAs, and EAAs, while several other candidate compounds showed fewer or no significant correlations. These patterns suggest co-variation among anatomical-cut composition and heated-headspace volatiles, but they do not establish formation pathways or causal metabolic regulation.

### 3.10. Limitations and Future Perspectives

Several limitations of this study should be acknowledged. First, the sample size of ten animals from a single commercial DLY population limits the generalizability of the findings. DLY pigs from different production systems, genetic lines, or geographical regions may exhibit different quality profiles. Second, volatile compound analysis was performed on six biological replicates per cut, which, although sufficient for exploratory profiling, limits the statistical power for detecting smaller differences. Third, the study was limited to a single breed (DLY) and did not include comparisons with indigenous Chinese pig breeds, which may exhibit different cut-specific quality characteristics. Fourth, the OAV-based aroma evaluation relies on odor thresholds determined in simplified model systems and does not account for matrix effects, compound interactions, or perceptual synergy. Fifth, no sensory evaluation or GC-olfactometry was performed, and the aroma-related conclusions should be considered as chemically inferred rather than sensory-validated. Finally, the volatile concentrations reported in this study are semi-quantitative estimates based on a single internal standard with RRF = 1.0.

Future studies should aim to: (i) expand the sample size and include multiple DLY populations and other pig breeds; (ii) validate the candidate discriminant aroma markers identified by OPLS-DA in an independent sample set; (iii) incorporate sensory evaluation and GC-olfactometry to validate the sensory relevance of the identified candidate aroma-relevant compounds; (iv) perform compound-specific calibration for accurate quantification of principal aroma compounds; and (v) evaluate the processing suitability of each cut through controlled cooking and processing experiments.

## 4. Conclusions

This exploratory within-animal study demonstrated distinct cut-related differences in the instrumental quality, nutritional composition, water distribution, and headspace volatile profiles of PJ, PB, BB, and SR obtained from a single commercial Duroc × Landrace × Yorkshire pig population. PB exhibited the lowest shear force, hardness, and chewiness, whereas BB had the highest crude protein, TAA, and EAA contents and the lowest crude fat content. PJ was characterized by the highest MUFA proportion and the lowest numerical AI and TI values, but it also had the highest n-6/n-3 PUFA ratio. Its higher cooking and centrifugal losses occurred together with the highest T_23_ and P23 values, consistent with lower water-retention ability under the present experimental conditions. SR contained the highest PUFA proportion and the greatest summed abundance of semi-quantified headspace volatiles.

Sixty-five volatile compounds were tentatively identified under the standardized 80 °C HS-SPME conditions. The OPLS-DA model differentiated the four cuts, and 10 compounds were retained as candidate discriminant aroma-relevant compounds according to the combined selection criteria. These candidates require validation in independent populations. Because the study involved 10 pigs from one commercial population, used six biological replicates for volatile profiling, and did not include sensory evaluation or GC–olfactometry, the findings should be regarded as preliminary cut-specific characterization rather than evidence of sensory superiority or processing suitability. Further studies incorporating larger populations, compound-specific volatile quantification, and sensory-directed analyses are required to confirm these differences and their practical relevance.

## Figures and Tables

**Table 1:** Instrumental quality parameters of different pork cuts.

Index	PJ	PB	BB	SR
Cooking loss (%)	26.35 ± 1.41 ^c^	18.39 ± 2.59 ^a^	20.19 ± 1.53 ^b^	16.07 ± 1.34 ^a^
Centrifugal loss (%)	28.07 ± 1.80 ^b^	18.31 ± 2.61 ^a^	20.71 ± 2.56 ^a^	17.68 ± 1.49 ^a^
pH_24h_	5.73 ± 0.06 ^a^	5.70 ± 0.00 ^a^	5.91 ± 0.05 ^a^	5.80 ± 0.10 ^a^
L*	64.53 ± 2.09 ^d^	44.44 ± 3.03 ^b^	35.18 ± 2.73 ^a^	49.99 ± 4.48 ^c^
a*	1.04 ± 0.37 ^a^	7.62 ± 2.53 ^b^	6.02 ± 1.40 ^b^	6.90 ± 1.10 ^b^
b*	16.09 ± 1.69 ^b^	14.8 ± 1.78 ^b^	9.54 ± 1.72 ^a^	16.79 ± 2.12 ^b^
Shear force (gf)	2406.00 ± 87.62 ^c^	1771.67 ± 19.43 ^a^	2031.00 ± 64.86 ^b^	2170.00 ± 48.59 ^b^
Hardness (gf)	3974.25 ± 224.23 ^d^	2333.00 ± 82.06 ^a^	3057.00 ± 34.77 ^b^	3465.33 ± 96.92 ^c^
Resilience	0.36 ± 0.01 ^d^	0.29 ± 0.01 ^b^	0.32 ± 0.01 ^c^	0.26 ± 0.01 ^a^
Cohesiveness	0.63 ± 0.01 ^c^	0.64 ± 0.01 ^c^	0.61 ± 0.01 ^b^	0.58 ± 0.01 ^a^
Springiness (mm)	3.91 ± 0.10 ^b^	4.06 ± 0.12 ^b^	3.71 ± 0.20 ^a^	3.95 ± 0.02 ^b^
Chewiness (gf·mm)	10,354.33 ± 194.36 ^d^	5602.33 ± 161.11 ^a^	6496.00 ± 206.02 ^b^	7847.33 ± 42.77 ^c^

Note: Values are expressed as mean ± SD (*n* = 10). PJ, pork jowl; PB, pork belly; BB, Boston butt; SR, spare ribs; pH_24h_, pH measured at 24 h post mortem; L*, lightness; a*, redness; b*, yellowness; gf, gram-force; mm, millimeter. Different superscript lowercase letters within the same row indicate significant differences among cuts (*p* < 0.05).

**Table 2:** Nutritional composition of different pork cuts.

Component	PJ	PB	BB	SR
Protein (%)	17.37 ± 0.45 ^ab^	17.40 ± 0.50 ^ab^	18.10 ± 0.40 ^b^	16.87 ± 0.78 ^a^
Fat (%)	17.20 ± 1.15 ^b^	20.37 ± 0.29 ^bc^	13.03 ± 0.90 ^a^	22.20 ± 1.01 ^c^
Carbohydrates (%)	0.54 ± 0.25 ^a^	0.47 ± 0.18 ^a^	0.52 ± 0.16 ^a^	0.49 ± 0.04 ^a^
Moisture (%)	63.97 ± 1.71 ^ab^	60.80 ± 0.56 ^a^	67.33 ± 0.31 ^b^	59.53 ± 0.21 ^a^
Ash (%)	0.93 ± 0.01 ^a^	0.96 ± 0.03 ^a^	1.02 ± 0.08 ^a^	0.91 ± 0.18 ^a^

Note: PJ, pork jowl; PB, pork belly; BB, Boston butt; SR, spare ribs. Different letters within the same row indicate a statistically significant difference (*p* < 0.05). *n* = 10.

**Table 3:** Amino-acid composition and content of different pork cuts on a wet-weight basis (g/100 g).

Amino Acid	PJ	PB	BB	SR
Isoleucine (Ile) *	0.87 ± 0.04 ^b^	0.89 ± 0.02 ^b^	0.95 ± 0.01 ^b^	0.74 ± 0.08 ^a^
Threonine (Thr) *	0.84 ± 0.03 ^b^	0.84 ± 0.04 ^b^	0.87 ± 0.03 ^b^	0.72 ± 0.04 ^a^
Serine (Ser)	0.73 ± 0.03 ^b^	0.72 ± 0.03 ^b^	0.73 ± 0.02 ^b^	0.59 ± 0.03 ^a^
Glutamic acid (Glu)	2.98 ± 0.16 ^b^	2.96 ± 0.10 ^b^	3.06 ± 0.04 ^b^	2.48 ± 0.08 ^a^
Glycine (Gly)	1.02 ± 0.12 ^b^	0.87 ± 0.03 ^ab^	0.87 ± 0.01 ^ab^	0.73 ± 0.05 ^a^
Alanine (Ala)	1.16 ± 0.06 ^b^	1.12 ± 0.05 ^b^	1.15 ± 0.06 ^b^	0.85 ± 0.01 ^a^
Valine (Val) *	0.88 ± 0.05 ^b^	0.89 ± 0.04 ^b^	0.93 ± 0.03 ^b^	0.75 ± 0.05 ^a^
Methionine (Met) *	0.07 ± 0.01 ^a^	0.15 ± 0.03 ^b^	0.07 ± 0.00 ^a^	0.10 ± 0.02 ^a^
Leucine (Leu) *	1.41 ± 0.06 ^b^	1.44 ± 0.06 ^b^	1.50 ± 0.02 ^b^	1.19 ± 0.06 ^a^
Tyrosine (Tyr)	0.57 ± 0.01 ^b^	0.59 ± 0.01 ^b^	0.62 ± 0.03 ^b^	0.41 ± 0.02 ^a^
Phenylalanine (Phe) *	0.78 ± 0.04 ^b^	0.79 ± 0.03 ^b^	0.84 ± 0.05 ^b^	0.62 ± 0.01 ^a^
Lysine (Lys) *	1.68 ± 0.02 ^b^	1.72 ± 0.03 ^b^	1.79 ± 0.03 ^c^	1.44 ± 0.02 ^a^
Histidine (His) *	0.63 ± 0.03 ^ab^	0.69 ± 0.03 ^ab^	0.72 ± 0.02 ^b^	0.59 ± 0.05 ^a^
Proline (Pro)	0.62 ± 0.03 ^b^	0.55 ± 0.03 ^ab^	0.54 ± 0.03 ^ab^	0.43 ± 0.03 ^a^
Arginine (Arg)	1.33 ± 0.07 ^b^	1.19 ± 0.05 ^ab^	1.22 ± 0.07 ^ab^	0.96 ± 0.07 ^a^
Aspartic acid (Asp)	1.67 ± 0.06 ^b^	1.68 ± 0.03 ^b^	1.71 ± 0.02 ^b^	1.40 ± 0.01 ^a^
Total amino acids (TAA)	17.26 ± 0.42 ^b^	17.09 ± 0.50 ^b^	17.58 ± 0.38 ^b^	13.99 ± 0.16 ^a^
Essential amino acids (EAAs)	7.16 ± 0.21 ^b^	7.41 ± 0.20 ^bc^	7.67 ± 0.15 ^c^	6.16 ± 0.17 ^a^
Non-essential amino acids (NEAAs)	10.09 ± 0.20 ^b^	9.68 ± 0.31 ^b^	9.91 ± 0.23 ^b^	7.84 ± 0.08 ^a^
EAAs/TAA	0.42 ± 0.00 ^a^	0.43 ± 0.00 ^b^	0.44 ± 0.00 ^b^	0.44 ± 0.01 ^b^
EAAs/NEAAs	0.71 ± 0.01 ^a^	0.77 ± 0.01 ^b^	0.77 ± 0.01 ^b^	0.79 ± 0.03 ^b^

Note: PJ, pork jowl; PB, pork belly; BB, Boston butt; SR, spare ribs. Different letters within the same row indicate a statistically significant difference (*p* < 0.05). * denotes essential amino acids (EAAs). TAA: total amino acids; NEAAs: non-essential amino acids. *n* = 10.

**Table 4:** Fatty-acid composition of different pork cuts (%).

Fatty Acid	PJ	PB	BB	SR
Capric acid (C10:0)	0.11 ± 0.01 ^a^	0.09 ± 0.01 ^a^	0.10 ± 0.00 ^a^	0.10 ± 0.01 ^a^
Lauric acid (C12:0)	0.09 ± 0.00 ^a^	0.08 ± 0.00 ^a^	0.08 ± 0.01 ^a^	0.09 ± 0.01 ^a^
Myristic acid (C14:0)	1.49 ± 0.05 ^a^	1.33 ± 0.03 ^a^	1.41 ± 0.05 ^a^	1.42 ± 0.18 ^a^
Palmitic acid (C16:0)	26.43 ± 1.00 ^a^	26.43 ± 0.46 ^a^	27.17 ± 0.61 ^a^	27.10 ± 0.70 ^a^
Margaric acid (C17:0)	0.14 ± 0.02 ^a^	0.18 ± 0.02 ^a^	0.14 ± 0.01 ^a^	0.16 ± 0.04 ^a^
Stearic acid (C18:0)	12.17 ± 0.49 ^a^	15.07 ± 0.81 ^b^	14.67 ± 0.40 ^b^	15.80 ± 1.20 ^b^
∑SFA	40.43 ± 1.51 ^a^	43.18 ± 1.28 ^b^	43.56 ± 1.04 ^b^	44.66 ± 0.33 ^b^
Palmitoleic acid (C16:1)	3.95 ± 0.20 ^b^	2.46 ± 0.06 ^a^	2.82 ± 0.14 ^a^	2.31 ± 0.36 ^a^
Trans oleic acid (C18:1, trans)	0.11 ± 0.02 ^b^	0.10 ± 0.00 ^ab^	0.10 ± 0.00 ^ab^	0.08 ± 0.01 ^a^
Oleic acid (C18:1)	44.93 ± 0.68 ^b^	41.93 ± 1.00 ^a^	40.90 ± 1.01 ^a^	39.83 ± 1.48 ^a^
Eicosenoic acid (C20:1)	0.79 ± 0.06 ^a^	0.79 ± 0.04 ^a^	0.86 ± 0.02 ^b^	0.85 ± 0.07 ^b^
Nervonic acid (C24:1)	0.14 ± 0.01 ^ab^	0.16 ± 0.00 ^b^	0.15 ± 0.02 ^ab^	0.12 ± 0.01 ^a^
∑MUFA	49.92 ± 0.85 ^b^	45.44 ± 1.09 ^a^	44.83 ± 1.17 ^a^	43.19 ± 1.77 ^a^
Linoleic acid (C18:2n6c)	8.03 ± 0.55 ^a^	8.99 ± 0.66 ^ab^	9.58 ± 0.31 ^ab^	9.90 ± 1.16 ^b^
γ-Linolenic acid (C18:3n6)	0.22 ± 0.13 ^a^	0.26 ± 0.00 ^b^	0.26 ± 0.01 ^b^	0.27 ± 0.02 ^b^
α-Linolenic acid (C18:3n3)	0.34 ± 0.02 ^a^	0.35 ± 0.03 ^a^	0.33 ± 0.01 ^a^	0.36 ± 0.05 ^a^
Eicosadienoic acid (C20:2)	0.37 ± 0.01 ^a^	0.41 ± 0.03 ^a^	0.44 ± 0.01 ^a^	0.46 ± 0.08 ^a^
Eicosatrienoic acid (C20:3n6)	0.13 ± 0.02 ^a^	0.14 ± 0.01 ^a^	0.10 ± 0.01 ^a^	0.10 ± 0.01 ^a^
Eicosatrienoic acid (C20:3n3)	0.04 ± 0.00 ^a^	ND	0.17 ± 0.24 ^a^	0.03 ± 0.03 ^a^
Arachidonic acid (C20:4n6)	0.41 ± 0.08 ^ab^	0.48 ± 0.04 ^b^	0.41 ± 0.06 ^ab^	0.31 ± 0.04 ^a^
Docosahexaenoic acid (DHA, C22:6n3)	0.14 ± 0.06 ^a^	0.74 ± 0.22 ^c^	0.48 ± 0.09 ^b^	0.75 ± 0.08 ^c^
∑PUFA	9.69 ± 0.72 ^a^	11.38 ± 0.93 ^ab^	11.77 ± 0.11 ^ab^	12.19 ± 1.35 ^b^
∑n-6	8.80 ± 0.65 ^a^	9.87 ± 0.71 ^a^	10.35 ± 0.28 ^a^	10.58 ± 1.23 ^a^
∑n-3	0.53 ± 0.07 ^a^	1.09 ± 0.24 ^b^	0.98 ± 0.26 ^b^	1.14 ± 0.09 ^b^
PUFA/SFA	0.24 ± 0.03 ^a^	0.26 ± 0.03 ^a^	0.27 ± 0.01 ^a^	0.27 ± 0.03 ^a^
n-6/n-3	16.81 ± 1.08 ^b^	9.27 ± 1.64 ^a^	11.07 ± 2.90 ^a^	9.27 ± 0.96 ^a^
AI	0.55 ± 0.03 ^a^	0.56 ± 0.02 ^ab^	0.59 ± 0.03 ^ab^	0.60 ± 0.02 ^b^
TI	1.29 ± 0.09 ^a^	1.38 ± 0.09 ^ab^	1.41 ± 0.09 ^ab^	1.46 ± 0.02 ^b^

Note: ND: not detected. Different letters within the same row indicate a statistically significant difference (*p* < 0.05). *n* = 10. PJ, pork jowl; PB, pork belly; BB, Boston butt; SR, spare ribs, ∑SFA, total saturated fatty acids; ∑MUFA, total monounsaturated fatty acids; ∑PUFA, total polyunsaturated fatty acids; AI, atherogenic index; TI, thrombogenic index, PUFA/SFA, ratio of total PUFA to total SFA; ∑n-6, total n-6 polyunsaturated fatty acids; ∑n-3, total n-3 polyunsaturated fatty acids; n-6/n-3, ratio of total n-6 PUFA to total n-3 PUFA.

**Table 5:** The T_2_ relaxation time and peak area proportions in different pork cuts.

Cut	T_21_ (ms)	T_22_ (ms)	T_23_ (ms)	P21 (%)	P22 (%)	P23 (%)
PJ	0.97 ± 0.07 ^b^	49.05 ± 2.43 ^a^	1076.92 ± 358.32 ^c^	3.49 ± 0.26 ^a^	95.43 ± 0.27 ^a^	1.08 ± 0.13 ^b^
PB	0.74 ± 0.14 ^a^	50.26 ± 1.71 ^a^	825.31 ± 95.58 ^b^	3.99 ± 0.25 ^b^	95.58 ± 0.20 ^a^	0.42 ± 0.12 ^a^
BB	1.43 ± 0.25 ^c^	52.34 ± 2.12 ^a^	731.21 ± 149.09 ^b^	3.71 ± 0.33 ^ab^	95.81 ± 0.31 ^a^	0.48 ± 0.21 ^a^
SR	0.69 ± 0.07 ^a^	49.97 ± 1.18 ^a^	440.18 ± 21.61 ^a^	3.92 ± 0.37 ^ab^	95.61 ± 0.34 ^a^	0.41 ± 0.26 ^a^

Note: Different letters in the same column indicate a statistically significant difference (*p* < 0.05). PJ, pork jowl; PB, pork belly; BB, Boston butt; SR, spare ribs. T_21_, T_22_, and T_23_ denote the relaxation times of the tightly associated, immobilized, and least-restricted water populations, respectively; P21, P22, and P23 denote their corresponding peak-area proportions.

**Table 6:** HS-SPME-GC-MS analysis results of volatile flavor compounds in different pork cuts. Concentrations are semi-quantitative estimates (μg/kg) calculated using an internal standard (2-methyl-3-heptanone) with a relative response factor (RRF) assumed to be 1.0.

Count	Compound Name	Formula	CAS	RT (min)	Concentration (μg/kg)			
					PJ	PB	BB	SR
Alcohols (28)								
1	2-Pentanol	C_5_H_12_O	6032-29-7	2.787	199.27 ± 37.92 ^a^	ND	301.40 ± 50.19 ^a^	1902.71 ± 199.30 ^b^
2	1-Pentanol	C_5_H_12_O	71-41-0	4.458	391.72 ± 64.26 ^a^	ND	1681.64 ± 248.31 ^b^	ND
3	1-Hexanol	C_6_H_14_O	111-27-3	8.152	54.35 ± 17.92 ^a^	146.25 ± 31.57 ^b^	153.07 ± 32.83 ^b^	212.78 ± 46.29 ^c^
4	1-Octen-3-ol	C_8_H_16_O	3391-86-4	13.192	1872.43 ± 427.18 ^a^	4474.27 ± 502.77 ^b^	1464.65 ± 264.16 ^a^	5277.83 ± 648.99 ^c^
5	3-Methylcyclopentanol	C_6_H_12_O	18729-48-1	14.045	ND	132.60 ± 13.18 ^a^	116.93 ± 18.08 ^a^	1237.42 ± 184.65 ^b^
6	(E)-3-Hexen-1-ol	C_6_H_12_O	928-97-2	14.298	1961.18 ± 311.95 ^a^	4338.91 ± 547.70 ^c^	ND	3421.37 ± 418.74 ^b^
7	2,2-Dimethyl-3-hexanol	C_8_H_18_O	4209-90-9	15.363	65.00 ± 19.43 ^c^	43.54 ± 11.65 ^b^	117.78 ± 18.15 ^d^	19.89 ± 2.76 ^a^
8	2-Nonen-1-ol	C_9_H_18_O	22104-79-6	15.663	947.97 ± 180.25 ^b^	317.27 ± 37.58 ^a^	327.73 ± 50.49 ^a^	1303.18 ± 131.39 ^c^
9	1-Octyn-3-ol	C_8_H_14_O	818-72-4	16.175	89.99 ± 12.46 ^a^	223.21 ± 31.36 ^b^	105.71 ± 21.75 ^a^	328.51 ± 37.94 ^c^
10	trans-1,2-Cyclopentanediol	C_5_H_10_O_2_	5057-99-8	16.504	24.85 ± 5.27 ^a^	81.73 ± 10.64 ^b^	24.16 ± 4.90 ^a^	101.65 ± 15.27 ^c^
11	2-Ethyl-1-hexanol	C_8_H_18_O	104-76-7	17.016	1490.36 ± 295.72 ^b^	1093.41 ± 118.23 ^a^	1258.92 ± 174.80 ^ab^	2210.16 ± 342.57 ^c^
12	3-Octen-2-ol	C_8_H_16_O	76649-14-4	17.645	212.54 ± 40.05 ^a^	656.04 ± 58.62 ^b^	202.32 ± 26.33 ^a^	845.28 ± 110.77 ^c^
13	1-Octanol	C_8_H_18_O	111-87-5	17.786	979.36 ± 203.76 ^b^	644.57 ± 95.13 ^a^	1014.81 ± 163.49 ^b^	2157.52 ± 195.27 ^c^
14	(E)-2-Nonen-1-ol	C_9_H_18_O	31502-14-4	19.028	4310.23 ± 705.67 ^a^	3703.97 ± 413.31 ^a^	5427.44 ± 856.17 ^a^	8530.15 ± 986.99 ^b^
15	3,7-Dimethyl-1-octanol	C_10_H_22_O	106-21-8	19.315	44.93 ± 27.93 ^a^	676.78 ± 78.62 ^b^	ND	1038.61 ± 200.21 ^c^
16	1-Decanol	C_10_H_22_O	112-30-1	21.198	23.28 ± 9.21 ^b^	56.93 ± 5.56 ^d^	13.47 ± 1.87 ^a^	42.18 ± 9.29 ^c^
17	4,8-Dimethyl-1-nonanol	C_11_H_24_O	33933-80-1	21.457	15.44 ± 4.76 ^a^	50.92 ± 4.39 ^b^	15.58 ± 3.30 ^a^	47.35 ± 5.08 ^b^
18	1-Undecanol	C_11_H_24_O	112-42-5	22.451	962.42 ± 174.62 ^c^	71.78 ± 6.08 ^a^	ND	195.67 ± 20.59 ^b^
19	2-Butyl-1-octanol	C_12_H_26_O	3913-02-8	22.733	64.08 ± 27.33 ^b^	9.81 ± 1.96 ^a^	2336.90 ± 441.18 ^d^	291.64 ± 32.78 ^c^
20	1-Dodecanol	C_12_H_26_O	112-53-8	24.515	ND	1332.73 ± 132.43 ^b^	ND	1105.46 ± 99.72 ^a^
21	11-Methyldodecanol	C_13_H_28_O	85763-57-1	25.68	21.88 ± 9.35 ^b^	14.34 ± 1.50 ^a^	21.69 ± 3.17 ^b^	29.57 ± 6.92 ^c^
22	7-Tetradecanol	C_14_H_30_O	3981-79-1	26.409	71.99 ± 20.91 ^b^	23.54 ± 2.84 ^a^	ND	24.32 ± 2.72 ^a^
23	trans-2-Undecen-1-ol	C_11_H_22_O	75039-84-8	28.468	ND	283.17 ± 45.70 ^c^	187.16 ± 29.95 ^b^	51.21 ± 9.18 ^a^
24	n-Heptadecanol-1	C_17_H_36_O	1454-85-9	33.033	28.34 ± 13.49	ND	ND	ND
25	1-Octadecanol	C_18_H_38_O	112-92-5	34.644	44.70 ± 15.38 ^b^	ND	18.85 ± 2.89 ^a^	ND
26	1-Eicosanol	C_20_H_42_O	629-96-9	37.991	9.73 ± 3.12 ^a^	62.22 ± 7.40 ^c^	30.87 ± 4.45 ^b^	150.50 ± 13.76 ^d^
27	Behenic alcohol	C_22_H_46_O	661-19-8	41.32	64.42 ± 27.14 ^a^	106.23 ± 22.10 ^b^	199.37 ± 52.99 ^c^	253.64 ± 26.59 ^d^
28	Cyclobutanol	C_4_H_8_O	2919-23-5	56.32	ND	ND	78.43 ± 22.05	ND
Total					13,950.44 ± 2557.18 ^a^	18,544.22 ± 2062.27 ^b^	15,098.87 ± 2422.68 ^a^	30,778.57 ± 3578.66 ^c^
Aldehydes (12)								
29	Pentanal	C_5_H_10_O	110-62-3	3.169	ND	ND	108.17 ± 20.50 ^a^	291.77 ± 53.84 ^b^
30	Octanal	C_8_H_16_O	124-13-0	3.77	113.20 ± 27.00 ^c^	85.78 ± 25.74 ^b^	ND	26.57 ± 4.50 ^a^
31	Hexanal	C_6_H_12_O	66-25-1	5.293	6003.55 ± 1049.86 ^a^	7368.82 ± 885.75 ^a^	11,361.71 ± 1801.04 ^b^	17,314.58 ± 2530.46 ^c^
32	Heptanal	C_7_H_14_O	111-71-7	9.399	939.05 ± 163.88 ^a^	1262.21 ± 117.25 ^b^	1243.10 ± 224.35 ^b^	1795.21 ± 171.37 ^c^
33	(E)-2-Heptenal	C_7_H_12_O	18829-55-5	11.987	990.49 ± 199.78 ^b^	1352.44 ± 154.57 ^c^	682.65 ± 122.45 ^a^	2088.66 ± 213.32 ^d^
34	Nonanal	C_9_H_18_O	124-19-6	18.457	55.97 ± 18.22 ^a^	771.74 ± 74.34 ^c^	ND	112.28 ± 11.79 ^b^
35	Decanal	C_10_H_22_O	112-31-2	20.163	46.35 ± 26.03 ^ab^	85.87 ± 13.06 ^c^	39.34 ± 7.23 ^a^	63.45 ± 6.00 ^b^
36	Undecanal	C_11_H_22_O	112-44-7	21.839	39.15 ± 12.07 ^b^	25.44 ± 6.18 ^a^	21.38 ± 11.72 ^a^	52.34 ± 5.76 ^c^
37	(E)-2-Nonenal	C_9_H_16_O	18829-56-6	22.016	10.14 ± 3.08 ^a^	768.15 ± 84.22 ^b^	ND	2372.60 ± 256.46 ^c^
38	2-Undecenal	C_11_H_20_O	2463-77-6	31.315	995.42 ± 203.56 ^c^	445.31 ± 70.41 ^b^	282.77 ± 61.59 ^a^	223.91 ± 36.32 ^a^
39	Pentadecanal	C_15_H_30_O	2765-11-9	45.044	236.66 ± 51.27 ^b^	54.63 ± 18.39 ^a^	188.70 ± 52.57 ^b^	204.64 ± 24.61 ^b^
40	Hexadecanal	C_16_H_32_O	629-80-1	48.591	294.46 ± 62.36 ^c^	63.49 ± 8.86 ^a^	356.06 ± 89.55 ^b^	211.16 ± 56.78 ^b^
Total					9724.42 ± 1731.9 ^a^	12,283.88 ± 1387.82 ^ab^	14,283.88 ± 2348.66 ^b^	24,757.15 ± 3266.39 ^c^
Acids (8)								
41	Hexanoic acid	C_6_H_12_O_2_	142-62-1	3.434	36.79 ± 8.24 ^a^	602.53 ± 247.92 ^b^	768.14 ± 125.19 ^b^	1540.61 ± 233.53 ^c^
42	2-Decenoic acid	C_10_H_18_O_2_	3913-85-7	23.88	67.45 ± 18.53 ^a^	282.48 ± 40.14 ^b^	ND	807.98 ± 128.27 ^c^
43	3-Propylglutaric acid	C_8_H_14_O_4_	4165-98-4	24.886	ND	21.32 ± 2.41 ^a^	18.32 ± 3.81 ^a^	ND
44	(E)-2-Tridecenoic acid	C_13_H_24_O_2_	32466-55-0	28.85	ND	51.08 ± 14.23 ^a^	20.88 ± 5.82 ^a^	1803.21 ± 209.48 ^b^
45	Myristoleic acid	C_14_H_26_O_2_	544-64-9	29.315	734.80 ± 153.89 ^b^	429.34 ± 65.31 ^a^	466.06 ± 75.50 ^a^	ND
46	n-Decanoic acid	C_10_H_20_O_2_	334-48-5	32.115	43.00 ± 13.41 ^a^	78.10 ± 23.71 ^a^	338.34 ± 53.82 ^b^	ND
47	8-Methylnonanoic acid	C_10_H_20_O_2_	5963-14-4	35.368	33.5 ± 11.88 ^a^	ND	72.19 ± 19.25 ^b^	ND
48	2-Methyl-octanoic acid	C_9_H_18_O_2_	3004-93-1	45.456	ND	ND	21.19 ± 5.63	ND
Total					915.54 ± 202.89 ^a^	1464.85 ± 298.32 ^b^	1705.67 ± 261.50 ^b^	4151.80 ± 512.55 ^c^
Esters (9)								
49	Hexanoic acid methyl ester	C_7_H_14_O_2_	106-70-7	10.557	102.02 ± 25.65 ^a^	843.91 ± 83.75 ^b^	ND	3302.73 ± 405.15 ^c^
50	Methyl nonanoate	C_10_H_20_O_2_	1731-84-6	20.38	24.07 ± 6.48 ^a^	34.22 ± 5.29 ^a^	3458.75 ± 519.21 ^c^	2266.81 ± 235.30 ^b^
51	Citronellyl isobutyrate	C_14_H_26_O_2_	97-89-2	24.663	211.18 ± 38.36 ^c^	90.59 ± 7.93 ^a^	87.34 ± 13.46 ^a^	128.20 ± 13.84 ^b^
52	Lauryl acetate	C_14_H_28_O_2_	112-66-3	26.798	1314.24 ± 216.94 ^a^	914.88 ± 132.74 ^a^	1065.45 ± 193.55 ^a^	ND
53	Pentanoic acid decyl ester	C_15_H_30_O_2_	5454-12-6	28.062	93.16 ± 19.63 ^b^	62.90 ± 6.80 ^a^	51.79 ± 8.26 ^a^	625.59 ± 55.24 ^c^
54	Methyl decanoate	C_11_H_22_O_2_	110-42-9	29.655	47.42 ± 18.40 ^a^	176.26 ± 28.48 ^ab^	1249.63 ± 209.17 ^c^	280.03 ± 28.01 ^b^
55	Dodecanoic acid ethyl ester	C_14_H_28_O_2_	106-33-2	40.703	90.63 ± 17.99 ^b^	105.73 ± 16.62 ^b^	180.10 ± 36.72 ^c^	54.95 ± 8.29 ^a^
56	Tetradecanoic acid ethyl ester	C_16_H_32_O_2_	124-06-1	47.879	ND	157.02 ± 23.55 ^b^	88.15 ± 11.80 ^a^	ND
57	Hexadecanoic acid ethyl ester	C_18_H_36_O_2_	628-97-7	54.684	64.27 ± 27.05 ^b^	ND	32.80 ± 4.42 ^a^	33.19 ± 6.47 ^a^
Total					1946.98 ± 346.59 ^a^	2385.51 ± 270.61 ^a^	6214.02 ± 973.57 ^b^	6691.50 ± 695.33 ^b^
Alkenes (4)								
58	6-Methyl-2-heptene	C_8_H_16_	73548-72-8	12.798	297.77 ± 42.93 ^a^	297.57 ± 71.83 ^a^	464.73 ± 86.07 ^a^	2037.54 ± 261.52 ^b^
59	(E)-1,3-Nonadiene	C_9_H_16_	56700-77-7	15.545	ND	149.95 ± 42.96	ND	ND
60	(Z)-3-Hexadecene	C_16_H_32_	34303-81-6	26.609	ND	23.46 ± 3.36 ^a^	ND	2518.9 ± 247.79 ^b^
61	Neophytadiene	C_20_H_38_	504-96-1	30.709	ND	164.62 ± 28.77	ND	ND
Total					297.77 ± 42.93 ^a^	635.60 ± 121.00 ^a^	464.73 ± 86.07 ^a^	4556.43 ± 501.99 ^b^
Ketones (1)								
62	2,3-Octanedione	C_8_H_14_O_2_	585-25-1	13.451	1874.10 ± 314.9 ^a^	5085.39 ± 597.95 ^c^	2285.77 ± 396.85 ^b^	4963.33 ± 612.64 ^c^
Total					1874.10 ± 314.9 ^a^	5085.39 ± 597.95 ^c^	2285.77 ± 396.85 ^b^	4963.33 ± 612.64 ^c^
Others (3)								
63	2-Pentylfuran	C_9_H_14_O	3777-69-3	13.657	891.10 ± 168.23 ^a^	1697.80 ± 175.99 ^c^	1010.50 ± 190.54 ^a^	1252.71 ± 179.68 ^b^
64	Anethole	C_10_H_12_O	104-46-1	27.939	57.31 ± 18.55 ^a^	70.06 ± 10.84 ^ab^	85.58 ± 16.90 ^b^	ND
65	6-Methyltridecane	C_14_H_30_	13287-21-3	22.21	9.46 ± 2.61 ^a^	92.11 ± 12.28 ^b^	ND	547.58 ± 55.91 ^c^
Total					957.87 ± 185.37 ^a^	1859.98 ± 194.97 ^b^	1096.08 ± 207.00 ^a^	1800.28 ± 233.26 ^b^

Note: PJ, pork jowl; PB, pork belly; BB, Boston butt; SR, spare ribs; a compound was classified as ND when the corresponding chromatographic signal had an S/N ratio < 3 or could not be reliably integrated. Different superscript lowercase letters indicate significant Tukey-adjusted pairwise differences among cuts for compounds with *p* < 0.05.

**Table 7:** Nominal OAV of tentatively identified headspace volatiles in different pork cuts.

Count	Compound Name	Odor Threshold (µg/kg) [27]	Odor Description	OAV			
				PJ	PB	BB	SR
1	2-Pentanol	10,000	Fermented, fine	0.02	ND	0.03	0.19
2	1-Pentanol	120	Malt aroma, fermented	3.26	ND	14.01	ND
3	1-Hexanol	150	Green	0.36	0.97	1.02	1.42
4	1-Octen-3-ol	7	Mushroom, earthy	267.49	639.18	209.24	753.98
5	3-Methylcyclopentanol	-	-	-	-	-	-
6	(E)-3-Hexen-1-ol	70	Green	28.02	61.98	ND	48.88
7	2,2-Dimethyl-3-hexanol	-	-	-	-	-	-
8	2-Nonen-1-ol	130	Green, fatty melon, waxy	7.29	2.44	2.52	10.02
9	1-Octyn-3-ol	-	-	-	-	-	-
10	trans-1,2-Cyclopentanediol	-	-	-	-	-	-
11	2-Ethyl-1-hexanol	300	Apple, pear	4.97	3.64	4.20	7.37
12	3-Octen-2-ol	-	-	-	-	-	-
13	1-Octanol	100	Fatty, citrus	9.79	6.45	10.15	21.58
14	(E)-2-Nonen-1-ol	130	Fatty, violet	33.16	28.49	41.75	65.62
15	3,7-Dimethyl-1-octanol	-	-	-	-	-	-
16	1-Decanol	0.7	Alcoholic, rose	33.26	81.32	19.24	60.25
17	4,8-Dimethyl-1-nonanol	-	-	-	-	-	-
18	1-Undecanol	700	Fatty, pineapple	1.37	0.10	ND	0.28
19	2-Butyl-1-octanol	20	Mild, soft floral	3.20	0.49	116.85	14.58
20	1-Dodecanol	6	Waxy	ND	222.12	ND	184.24
21	11-Methyldodecanol	**-**	-	-	-	-	-
22	7-Tetradecanol	**-**	-	-	-	-	-
23	trans-2-Undecen-1-ol	-	-	-	-	-	-
24	1-Heptadecanol	-	-	-	-	-	-
25	1-Octadecanol	-	-	-	-	-	-
26	1-Eicosanol	-	-	-	-	-	-
27	Behenic alcohol	300	Waxy, fatty	0.21	0.35	0.66	0.85
28	Cyclobutanol	5000	Alcoholic, woody	ND	ND	0.02	ND
29	Pentanal	12	Nutty, bread	ND	ND	9.01	24.31
30	Octanal	0.8	Fatty, citrus	141.5	107.23	ND	33.22
31	Hexanal	4500	Green, apple	1.33	1.64	2.52	3.85
32	Heptanal	3	Fatty, citrus	313.02	420.74	414.37	598.4
33	(E)-2-Heptenal	60	Fruit, fatty	16.51	22.54	11.38	34.81
34	Nonanal	1	Fatty, green	55.97	771.74	ND	112.28
35	Decanal	3	Green, sweet	15.45	28.62	13.11	21.15
36	Undecanal	5	Waxy, citrus, rose	7.83	5.09	4.28	10.47
37	(E)-2-Nonenal	1	Fatty, green	10.14	768.15	ND	2372.6
38	2-Undecenal	-	-	-	-	-	-
39	Pentadecanal	1000	Fatty, citrus	0.24	0.05	0.19	0.20
40	Hexadecanal	500	Waxy	0.59	0.13	0.71	0.42
41	Hexanoic acid	3000	Butter, fermented	0.01	0.20	0.26	0.51
42	2-Decenoic acid	10,000	Fermented, fruit	0.01	0.03	ND	0.08
43	3-Propylglutaric acid	-	-	-	-	-	-
44	(E)-2-Tridecenoic acid	-	-	-	-	-	-
45	Myristoleic acid	-	-	-	-	-	-
46	n-Decanoic acid	10,000	Wine, coconut	<0.01	0.01	0.03	ND
47	8-Methylnonanoic acid	-	-	-	-	-	-
48	2-Methyl-octanoic acid	-	-	-	-	-	-
49	Hexanoic acid methyl ester	70	Fruit	1.46	12.06	ND	47.18
50	Methyl nonanoate	40	Wine, coconut	0.60	0.86	86.47	56.67
51	Citronellyl isobutyrate	10	Citrus, fruit	21.12	9.06	8.73	12.82
52	Lauryl acetate	410	Waxy, rose	3.21	2.23	2.60	ND
53	Pentanoic acid decyl ester	-	-	-	-	-	-
54	Methyl decanoate	5	Wine, fruit	9.48	35.25	249.93	56.01
55	Dodecanoic acid ethyl ester	400	Butter, fruit	0.23	0.26	0.45	0.14
56	Tetradecanoic acid ethyl ester	500	Iris-like, sweet	ND	0.31	0.18	ND
57	Hexadecanoic acid ethyl ester	2000	Butter, fatty	0.03	ND	0.02	0.02
58	6-Methyl-2-heptene	400	Alcoholic	0.74	0.74	1.16	5.09
59	(E)-1,3-Nonadiene	8400	Butter, rancid	ND	0.02	ND	ND
60	(Z)-3-Hexadecene	-	-	-	-	-	-
61	Neophytadiene	-	-	-	-	-	-
62	2,3-Octanedione	29	Butter, fatty	64.62	175.36	78.82	171.15
63	2-Pentylfuran	6	Fruit, vegetable	148.52	282.97	168.42	208.78
64	Anethole	20	Anise, licorice	2.87	3.5	4.28	ND
65	6-Methyltridecane	-	-	-	-	-	-

Note: PJ, pork jowl; PB, pork belly; BB, Boston butt; SR, spare ribs; OAV = concentration of compound ÷ odor threshold. Odor thresholds were retrieved from Van Gemert [27]. “-” = odor threshold not available in the published literature. “ND”, not detected based on S/N < 3 or the absence of a reliably integrated peak.

## Data Availability

The original contributions presented in this study are included in the article. Further inquiries can be directed to the corresponding authors.
